# Cost-Effectiveness of Olaratumab in Combination with Doxorubicin for Patients with Soft Tissue Sarcoma in the United States

**DOI:** 10.1155/2018/6703963

**Published:** 2018-03-26

**Authors:** Santiago Zuluaga-Sanchez, Lisa M. Hess, Sorrel E. Wolowacz, Yulia D'yachkova, Emma Hawe, Adrian D. Vickers, James A. Kaye, David Bertwistle

**Affiliations:** ^1^RTI Health Solutions, 2nd Floor, The Pavilion, Towers Business Park, Wilmslow Road, Didsbury, Manchester M20 2LS, UK; ^2^Eli Lilly and Company, Lilly Corporate Center, Indianapolis, IN 46285, USA; ^3^Eli Lilly GmbH, Koelblgasse 8, 1030 Vienna, Austria; ^4^RTI Health Solutions, 1440 Main Street, Suite 310, Waltham, MA 02451, USA; ^5^Eli Lilly and Company Limited, Erl Wood Manor, Sunninghill Road, Windlesham, Surrey GU20 6PH, UK

## Abstract

**Background:**

Standard first-line treatments for advanced soft tissue sarcoma (STS) have changed little for 40 years, and outcomes have been poor. Recently, the United States (US) Food and Drug Administration conditionally approved olaratumab in combination with doxorubicin (Olara + Dox) based on a randomized phase II trial that reported a significant 11.8-month improvement in median survival versus single-agent doxorubicin (Dox). The present study investigated the cost-effectiveness of Olara + Dox compared with Dox and five other standard-of-care regimens from the US payer perspective.

**Methods:**

An economic model was constructed to estimate costs and outcomes over patients' lifetimes from start of therapy. Progression-free and overall survival were based on survival analysis of patient-level data and a meta-analysis. Adverse-event rates were based on trials. Costs were from published sources.

**Results:**

Olara + Dox resulted in an estimated additional 1.27 life-years (LYs) compared with Dox, with an increase in total expected lifetime costs of $133,653. The incremental cost-effectiveness ratio (ICER) was estimated at $105,408 per LY gained; in a fully incremental analysis, all other regimens were dominated (higher costs and lower LYs or a higher ICER).

**Conclusion:**

Olara + Dox is cost-effective for STS treatment compared with Dox and other standard-of-care regimens at willingness-to-pay thresholds of $150,000 per LY and above.

## 1. Introduction

Soft tissue sarcomas (STSs) are a group of rare and heterogeneous solid tumors of mesenchymal cell origin with distinct clinical and pathologic features [1]. Together, STSs account for less than 1% of all new human malignant neoplasms [2, 3]. It is estimated that 12,310 people in the United States (US) were diagnosed with STS in 2016, and in the same year, there were approximately 4,990 deaths [4]. There are more than 50 subtypes of STS, including leiomyosarcoma, undifferentiated pleomorphic sarcoma, synovial sarcoma, liposarcomas, and malignant peripheral nerve sheath tumors [5]. Prognosis depends on several factors, including a patient's age and the location, size, depth, histologic grade, and stage of the tumor [1]. For most patients with advanced (locally advanced or metastatic) STS, the prognosis for long-term survival is poor; data from the US National Cancer Institute's Surveillance, Epidemiology, and End Results (SEER) program suggest that overall survival (OS) for metastatic STS (mSTS) patients is 48% at 1 year and 18% at 5 years [6].

Chemotherapy is recommended by the National Comprehensive Cancer Network [7] for the treatment of advanced disease, where the aim is to prolong survival and preserve organ function. A variety of agents are available for use in the US, both as single agent (e.g., ifosfamide (Ifo), dacarbazine, and doxorubicin (Dox)) and in combination (e.g., Dox or epirubicin with Ifo and/or dacarbazine) [7]. Until 2016, only Dox, Ifo, and epirubicin (alone or in combination) had shown significant response rates as first-line treatments [8]; patient survival remained poor, with a median OS of 8 to 16 months for first- or second-line palliative chemotherapy [9–13]. Moreover, the standard of care for first-line therapy has changed little for 40 years [12, 14]. Other chemotherapeutic agents such as gemcitabine, docetaxel, vinorelbine, pegylated liposomal doxorubicin (PLD), and temozolomide have been evaluated in clinical trials; there is no clear evidence to support their use as optimal management of patients with advanced STS [7].

Olaratumab (Olara) (Lartruvo; Eli Lilly and Company) is a fully human immunoglobulin G1 monoclonal antibody that selectively binds the external domain of human platelet-derived growth factor receptor-α with high affinity and blocks ligand binding. In October 2016, the US Food and Drug Administration (FDA) granted accelerated approval to olaratumab in combination with doxorubicin (Olara + Dox) for the treatment of adult patients with STS with a histologic subtype for which an anthracycline-containing regimen is appropriate and not amenable to curative treatment with radiotherapy or surgery [15]. The European Medicines Agency reviewed olaratumab under its accelerated assessment program and granted conditional marketing authorization shortly after the FDA, in November 2016 [16]. Both regulatory submissions were based on the randomized phase 1b/2 trial JGDG [17], which reported a significant improvement in survival for Olara + Dox compared with single-agent Dox in the treatment of advanced STS (excluding Kaposi sarcoma and gastrointestinal stromal tumor) not amenable to curative treatment with surgery or radiotherapy. The primary endpoint of the phase 2 portion of the JGDG trial was progression-free survival (PFS), with secondary endpoints of OS and objective response rate. The confirmatory phase 3 trial evaluating the benefit/risk ratio for Olara + Dox is currently underway (NCT02451943, ANNOUNCE; Eli Lilly and Company [18]).

The National Comprehensive Cancer Network guidelines include Olara in combination with doxorubicin for treating histologic subtypes of STS for which an anthracycline-containing regimen is appropriate [7]. This research investigated the cost-effectiveness of Olara + Dox versus standard regimens for the treatment of adult patients with anthracycline-naïve, advanced STS not amenable to curative treatment with surgery or radiotherapy (the population studied in the JGDG trial). The analysis was performed from the US private payer perspective.

## 2. Methods

### 2.1. Model Population and Treatments

The model population included adult patients with anthracycline-naïve advanced STS who are not candidates for curative treatment with surgery or radiotherapy and who are to be treated with a first or subsequent line of systemic therapy. Patients with Kaposi sarcoma or gastrointestinal stromal tumor were not included in the population (consistent with the patient sample in the JGDG trial). Patient characteristics were from the JGDG trial [17]. In the model, Olara + Dox was compared with alternative regimens that (1) are currently licensed for use in the US for the treatment of advanced STS, (2) are recommended by the National Comprehensive Cancer Network guidelines [7], or (3) have been directly compared with Olara + Dox in a randomized trial or can be indirectly compared using conventional methods of network meta-analysis. These comparators were Dox (based on the JGDG trial [17]), Dox in combination with Ifo and mesna (AIM) (based on Judson et al. [12]), and gemcitabine in combination with docetaxel (GemDoc; based on the GeDDiS trial, hereafter referred to as GemDoc (GeDDiS) [19]). In addition to these comparators, three regimens that are used in routine practice also were compared: mesna in combination with Dox, Ifo, and dacarbazine (MAID) [20], an alternative GemDoc dosing schedule reported by Maki et al. [21] (hereafter referred to as GemDoc (Maki)) and PLD [22]. The trials investigating these three regimens did not connect with the randomized trial evidence network for Olara + Dox, Dox, AIM, and GemDoc (GeDDiS); therefore, assumptions (detailed in PFS and OS Estimates) were made with regard to their relative efficacy. The dosages, route of administration, and planned durations of therapy for the interventions included in the economic model are presented in Table 1.

### 2.2. Economic Model

**Figure 1:**
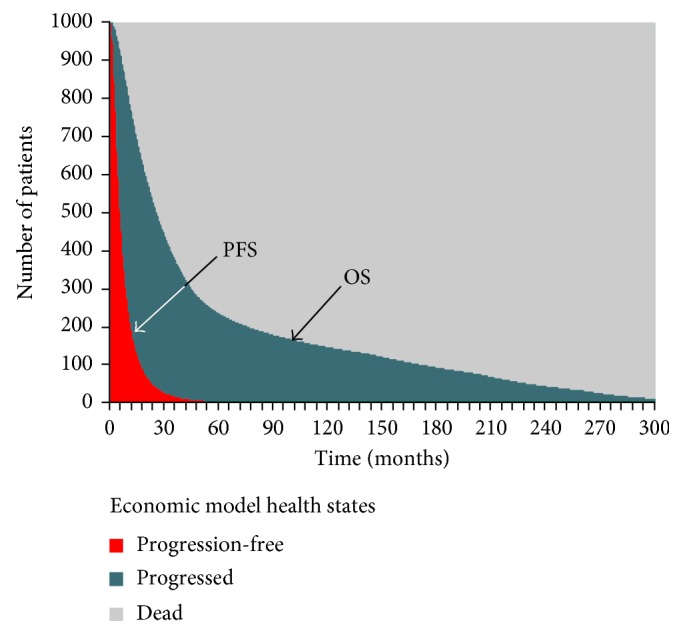
Economic model structure. OS = overall survival curve; PFS = progression-free survival curve. Note that the PFS and OS curves shown are illustrative only.

All aspects of this economic analysis followed standard best practices as published by the International Society for Pharmacoeconomics and Outcomes Research [25] and as recommended by health technology assessment bodies, such as the National Institute for Health and Care Excellence [26]. The model used a partitioned survival structure (Figure 1); selection of this structure was informed by clinical expert input and previous economic models in oncology (e.g., [27]). The model contained three health states: progression-free, progressed, and dead. The proportion of the patient cohort in each heath state over time was calculated from PFS and OS curves. The model time horizon was 25 years, which was sufficient to capture the predicted lifetime of all patients in the cohort from treatment initiation to death. This time horizon allowed estimation of the expected clinically important differences in costs and outcomes between patients receiving alternative systemic therapies for advanced STS. Model time was divided into increments (model cycles). At the end of each time increment, the proportion of patients in each health state was counted, as well as the costs incurred and the life-years (LYs) and quality-adjusted life-years (QALYs) accrued. A model cycle length of 1 week was used in order to ensure that differences in time to progression could be precisely modeled (median PFS was 6.6 months for Olara + Dox and 4.1 months for Dox in the JGDG trial [17]) as well as longer-term events such as death.

Patients entered the model at the initiation of systemic therapy (either Olara + Dox or a comparator) and were assigned to the Progression-free health state. Patients continued therapy until the completion of the planned regimen, disease progression, unacceptable toxicity, or discontinuation of therapy for another reason (e.g., patient or physician decision). In each model cycle, patients were partitioned into the progression-free health state, the progressed health state, or the dead state. Patients with disease progression could have further lines of active systemic therapy or could receive best supportive care only. As the cycle length was short in relation to the overall model time horizon, no half-cycle correction was applied [28].

Estimated health outcomes and costs were discounted in the model, using an annual discount rate of 3% [29].

The model inputs were identified by systematic literature reviews for clinical evidence [23] and economic studies (see Supplementary Materials (available here)) and were reviewed by clinical experts. The model input parameters are summarized in Tables 1 and 2 and in Supplementary Materials. Details of the PFS and OS inputs; adverse-event (AE) data; and health-related quality of life (utility), resource use, and cost estimates are provided in the following sections.

### 2.3. PFS and OS Estimates

For the direct comparison of Olara + Dox with Dox, a range of parametric survival functions (exponential, Weibull, log-normal, log-logistic, gamma, and Gompertz) were fitted to the patient-level data for PFS (investigator-assessed and blinded independent radiological review) and OS from the JGDG trial according to recognized guidelines [34]. Functions were fitted to data for both treatment arms together (using treatment as an indicator), as well as to each treatment arm separately. Survival models were fitted with covariates to allow for possible subgroup analysis and optimize the model fit to the data. Model fit to the observed data was evaluated using Akaike information criterion (AIC) and Bayesian information criterion (BIC) statistics and the visual fit of the function to the Kaplan–Meier data [34].

**Figure 2:**
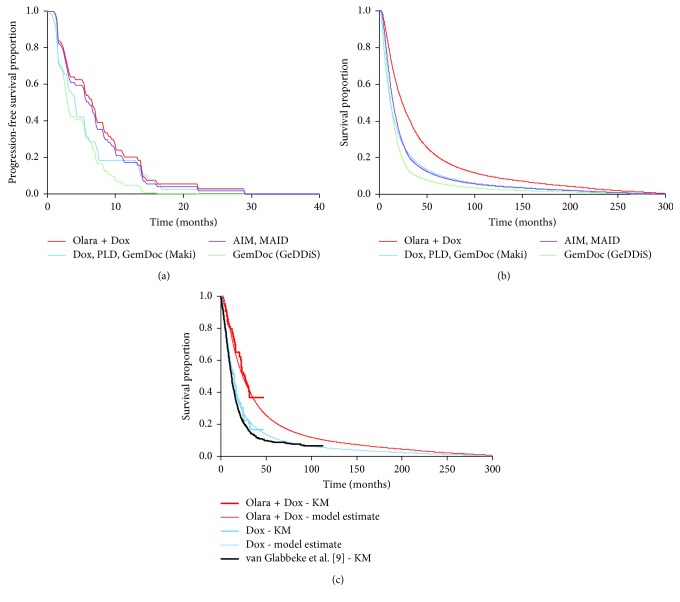
Survival estimates. (a) Progression-free survival; (b) overall survival; (c) overall survival validation. For the parametric survival models for PFS and OS, the following covariates were significant in most of the survival models explored and were included in the final set of survival models: tumor type and line of therapy and (where treatment was included as an indicator) the interaction of treatment with tumor type and line of therapy. For OS, the following additional covariates were significant in most models and were also included as covariates in the final analyses: sex and Eastern Cooperative Oncology Group performance status. AIM = ifosfamide + doxorubicin + mesna; Dox = doxorubicin; GemDoc = gemcitabine + docetaxel; MAID = mesna + doxorubicin + ifosfamide + dacarbazine; KM = Kaplan–Meier; Olara + Dox = olaratumab + doxorubicin; PLD = pegylated liposomal doxorubicin.

For PFS, none of the parametric functions were able to replicate the observed convergence of the Kaplan–Meier curves for Olara + Dox and Dox at approximately 12 months (Figure 2(a)). Since the Kaplan–Meier curves for PFS have essentially reached zero within the trial follow-up period (Figure 2(a)), extrapolation was not necessary to predict PFS beyond the end of the trial. Therefore, the JGDG Kaplan–Meier data were used directly to estimate PFS over time for Olara + Dox and Dox in the base-case economic analysis. In order to explore the uncertainty in the cost-effectiveness results arising from alternative approaches to modeling PFS, survival models fitted to each treatment arm separately were evaluated as scenario analyses. The investigator assessment of PFS was selected for the base case rather than the blinded independent review assessment, as investigator assessment more closely reflects assessment of disease progression in clinical practice which leads to discontinuation of therapy and possible initiation of subsequent lines of treatment. The blinded independent review assessment of PFS was explored as a scenario analysis.

Since the OS Kaplan–Meier curves from JGDG end in plateaus and do not reach zero during the 47 months of trial follow-up, extrapolation of the OS curves beyond the follow-up period was required in order to estimate health outcomes and costs over the entire lifetime of all patients. For OS, the proportional hazards assumption appeared reasonable (based on assessment of the Kaplan–Meier curves, log cumulative hazard plots, and proportional hazards test *P* value = 0.226). Therefore parametric functions fitted to both treatment arms together were used for the base-case economic analysis. The extrapolated portion of the OS curve for Dox in the economic model was validated by comparison with published long-term OS data for advanced STS patients receiving anthracycline-containing therapy. The most appropriate long-term OS data identified for this validation were those reported by van Glabbeke et al. [9] and consisted of almost 10 years of OS data from a meta-analysis of 2,185 patients in seven trials investigating anthracycline-based first-line therapy in advanced STS. Of the parametric functions explored, only the gamma function produced a plausible prediction for OS beyond the end of the JGDG trial data in the Dox arm, compared with the van Glabbeke data. This function was therefore used for the base-case analysis. The parameters for the gamma function for OS are presented in Table 1. Three other functions (log-normal, Weibull and Gompertz) were used in scenario analyses to explore the uncertainty in the cost-effectiveness results arising from alternative approaches to modeling OS. A range of assumptions regarding the continuation of treatment effect (assuming no treatment effect after the trial, the treatment effect tapers over a period of time, or continues indefinitely) were explored as scenario analyses. The base-case analysis assumed no treatment effect beyond 32 months (the time of last observed death in the Olara + Dox arm of the JGDG trial) by using the same hazard of death for each subsequent cycle in the Olara + Dox arm as in the Dox arm. Furthermore, the base case incorporated the increased risk of death from other causes with age (using US mortality rates; Centers for Disease Control and Prevention [35]), adjusted for patients with STS (estimated by comparison of the risk at the end of the Kaplan–Meier curve reported by van Glabbeke et al. [9], with the risk for the general population of the same age).  Figure 2(b) shows the resulting OS functions for Olara + Dox and the comparator regimens; Figure 2(c) compares the functions for Olara + Dox and Dox with the Kaplan–Meier OS data from the JGDG trial and the long-term OS data reported by van Glabbeke et al. [9].

In the JGDG trial, 30 of the 67 patients (45%) randomized to Dox received single-agent olaratumab after disease progression. The efficacy of single-agent olaratumab has not been studied in this setting, and it is not expected to be used in routine practice; therefore, a range of analyses were performed to explore the potential impact of its use in the JGDG trial on the OS outcomes observed. Three methods were applied (inverse probability of censoring weights, a simplified two-stage method, and methods using external data) as described by Latimer and colleagues [36, 37]. These analyses found no evidence that single-agent olaratumab use in the control arm after disease progression biased the results toward a larger study OS treatment effect. In addition, analysis results presented by Tap et al. [17] in their Supplementary Materials found that censoring OS at the time of starting any new anticancer therapy (HR: 0.425; 95% CI: 0.193–0.933), or a group of specific cancer therapies (pazopanib, eribulin, GemDoc, Dox, or trabectedin) (HR: 0.353; 95% CI: 0.192–0.647) produced similar HRs to the primary analysis of JGDG (HR 0.463; 95% CI 0.301–0.710). Therefore, no adjustment to the OS data was made in the economic model.

For the indirect comparisons with AIM and GemDoc (GeDDiS), HRs for Olara + Dox versus each regimen (for PFS and OS) were estimated via a network meta-analysis (NMA) [23]. The trials in the NMA for AIM and GemDoc (GeDDiS) investigated first-line therapy only, while the trial for Olara + Dox (the JGDG trial) investigated any line of therapy. The results of the NMA are shown in Table 1. Since PLD, GemDoc (Maki), and MAID could not be connected to the evidence network via randomized controlled trials [23], the indirect comparisons of Olara + Dox with these regimens were based on assumptions regarding their relative efficacy. PLD and GemDoc (Maki) were assumed to have the same efficacy as Dox, and MAID was assumed to have the same efficacy as AIM (Table 1). The HR for the indirect comparisons was applied in the economic model by calculating the instantaneous hazard for Olara + Dox in each model cycle from the survival functions, multiplying the result by the HR for each Olara + Dox comparator to generate the instantaneous hazard for the comparator, and finally calculating the survivor proportion in each model cycle for the comparator. The model assumed that after 32 months the hazards for death for all interventions were the same as those for Dox (consistent with trial data for AIM [12]; GemDoc (GeDDiS) [19]; and assumptions for MAID (assumed the same as AIM), PLD, and GemDoc (Maki) (assumed the same as Dox)).

### 2.4. Adverse Events

The incidence of each AE across the included trials (see Supplementary Materials) was obtained from the clinical trials referenced in Table 1; where specific AEs were not reported, the incidence was assumed to be zero. In order to account for instances of individual patients experiencing more than one episode of a given AE, the model used the mean number of AEs per patient having that AE as well as the incidence to calculate the total number of AEs (see Supplementary Materials). Costs for grade 3 or 4 AEs and (in a secondary analysis) the impact of any grade AEs on quality of life (health utility) were accounted for in the model (see Supplementary Materials).

### 2.5. Health-Related Quality of Life (Utility) Estimates

The model included a secondary analysis evaluating the cost utility of Olara + Dox (i.e., estimating the incremental cost per QALY gained). For this analysis, the model used published health utility estimates identified by a systematic literature review (see Supplementary Materials). In selecting data for the model from the available estimates, data that had been collected via a validated multiattribute utility instrument and estimated using a preference-based value set or by mapping from a validated health-related quality-of-life measure were preferred. The health utility estimates applied for the model health states are summarized in Supplementary Materials.

### 2.6. Resource Use and Costs

The model used unit costs for 2016; costs reported for other cost years were inflated to 2016 values using the consumer price index [38]. Unit costs were taken from recognized national sources, where available (i.e., Healthcare Cost and Utilization Project [39] and the Essential RBRVS [31]). Drug and drug-administration costs for Olara + Dox and Dox were calculated based on the mean dose per administration and the mean number of administrations for each drug as observed in the JGDG trial (thereby accounting for dose reductions and dose delays as they occurred in the JGDG trial). There was no need for prediction of treatment costs beyond trial follow-up, as all patients in the JGDG trial had discontinued their randomized treatment before the end of the study. For other interventions, planned doses were used as mean doses were not reported in the source trials (a sensitivity analysis explored dose reductions equivalent to that observed for Dox in the JGDG study); the mean number of administrations were based on trial data (where available) or assumptions (Table 1). AIM was assumed to be administered in an outpatient setting. Drug costs assumed that unused drug in opened vials is wasted. AIM and GemDoc (Maki) were assumed to be given along with pegfilgrastim. In order to account for the possibility that the less costly G-CSF filgrastim could be used, a sensitivity analysis was performed which assumed that the cost of G-CSF given with AIM and GemDoc (Maki) was zero (Supplementary Materials). Monitoring costs associated with cardiac monitoring for patients receiving Dox either with or without other agents, routine follow-up visits, and imaging also were included (Table 2). Additionally, the model incorporated the cost of subsequent lines of active systemic treatment, based on the treatments received in Olara + Dox arm of the JGDG trial after the investigational therapy (Table 2). Because some patients in the Dox arm received single-agent olaratumab after disease progression which is not expected to be used in clinical practice, the cost per subsequent treatment in the Dox arm was assumed to be the same as the average cost per subsequent treatment in the Olara + Dox arm.

### 2.7. Sensitivity and Scenario Analyses

One-way (univariate) sensitivity analyses were performed by varying all parameters individually to identify those that had the most influence on the incremental cost-effectiveness ratio (ICER). A probabilistic sensitivity analysis was performed in which the mean values for all model parameters were sampled simultaneously from their statistical distributions. The parameter uncertainties were based on estimates of uncertainty in the source data (e.g., the reported standard error), where available. Parameters were sampled from appropriate statistical distributions [40]. Additional scenario analyses were performed to investigate anticipated areas of particular uncertainty, for example, discount rates for costs and outcomes and alternative approaches to model PFS and OS (see Supplementary Materials).

### 2.8. Model Validation

Model validation was performed in alignment with best practices published by the International Society for Pharmacoeconomics and Outcomes Research [25]. Face validity of the model structure and input parameters was ensured by expert reviews involving a consultant medical oncologist in the UK, a medical oncologist professor in France, a medical oncologist in the US, 2 independent academic health economics and statistics experts in the UK, and an advisory board consisting of 10 clinical experts in the UK. Internal validity was assured by checks of input data and coding by a researcher not involved with the original model programming. Cross-validity (comparison of results with other models analyzing the same decision problem) could not be performed because no suitable studies were identified. Dependent external validity (comparing model predictions with outcomes in studies used to build the model, i.e., the JGDG trial) and independent external validity (comparing model predictions with outcomes in studies not used to build the model) were performed. These results are presented in Figure 2. Figure 2(a) shows PFS in the model. For Olara + Dox and Dox, PFS over time was estimated directly from the Kaplan–Meier data from the JGDG trial and therefore was identical to that in the trial (to the nearest weekly model cycle). Figure 2(c) compares OS for Olara + Dox and Dox in the model with the Kaplan–Meier OS data from the JGDG trial, as well as external long-term OS data reported by van Glabbeke et al. [9]. Validation of the final economic model by two independent health economics groups was also performed.

## 3. Results

### 3.1. Base-Case Results

The base-case results for the comparison of Olara + Dox, Dox, AIM, GemDoc (GeDDiS), GemDoc (Maki), PLD, and MAID are presented in Table 3. The base-case results indicated that the mean total expected lifetime cost was higher for patients receiving Olara + Dox than for those receiving the alternative active systemic therapies ($182,984 compared with $122,166 for AIM, $104,787 for MAID, $83,473 for GemDoc (Maki), $53,925 for PLD, $50,976 for GemDoc (GeDDiS), and $49,330 for Dox). Mean life-years (LYs) were increased substantially for patients receiving Olara + Dox; the base-case estimates for mean (discounted) LYs were 3.37 for Olara + Dox, compared with 2.17 for AIM and MAID; 2.10 for Dox, PLD, and GemDoc (Maki); and 1.69 for GemDoc (GeDDiS). The incremental cost per LY saved for Olara + Dox versus Dox was estimated as $105,408.

In pairwise comparisons, GemDoc (GeDDiS), PLD, and GemDoc (Maki) were dominated (a dominated intervention is defined as an intervention with higher costs and worse outcomes than an alternative intervention) by Dox because the total mean lifetime costs were higher while LYs were the same or lower than for Dox. GemDoc (GeDDiS) showed a median OS (estimated by applying the HR from the NMA to the gamma function used for Olara + Dox) that was slightly higher than Dox but had a steeper decline in OS, resulting in a lower mean OS estimate (Figure 2(b)). To determine which of the regimens under consideration was the most cost-effective, a fully incremental analysis was conducted. In a fully incremental analysis, a treatment is said to be extendedly dominated when the treatment's ICER is higher than the ICER of the next, more effective, alternative (i.e., the given treatment is dominated by the combination of two alternatives and should not be used to calculate appropriate ICERs). For example, consider that there are three drug regimens, A, B, and C, with regimen C being more effective (resulting in greater LYs) and more costly than regimen B, and regimen B being more effective (resulting in greater LYs) and more costly than regimen A. Drug regimen C is said to extendedly dominate drug regimen B if the ICER for drug regimen C when compared with drug regimen A is more favorable (has a lower value) than the ICER for drug regimen B when compared with drug regimen A. In this analysis, AIM was dominated by MAID because AIM's efficacy was assumed to be the same, while MAID was associated with lower costs (owing to the lower drug doses and the omission of granulocyte-colony stimulating factor). Olara + Dox was superior in cost-effectiveness to MAID because the ICER for the comparison between MAID and Dox was higher than the ICER for the comparison between Olara + Dox and Dox (MAID therefore was extendedly dominated by Olara + Dox).

In the secondary analysis (using QALYs as the outcome measure) Olara + Dox was associated with a mean (discounted) 1.86 QALYs gained, compared with 1.17 for Dox, PLD, and GemDoc (Maki); 0.94 for GemDoc (GeDDiS); and 1.21 for AIM and MAID. The incremental cost per QALY gained for Olara + Dox versus Dox was estimated as $196,309.

### 3.2. One-Way Sensitivity Analysis

**Figure 3:**
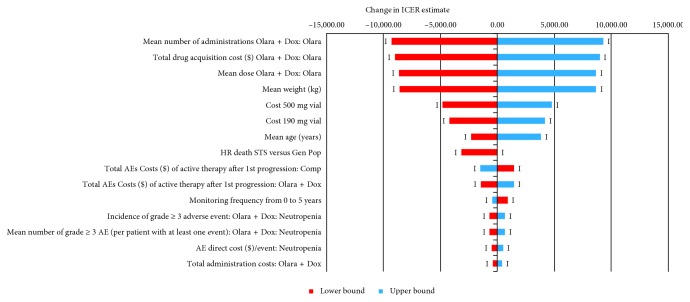
One-way sensitivity analysis tornado diagram for Olara + Dox versus Dox: change in ICER (US $ per LY saved). In the one-way sensitivity analysis, all model parameters were varied individually by +/− 10% of the base-case value with the exception of HR death STS versus Gen Pop, which was changed to 1.00. The plot shows the change in the incremental cost per LY gained for each parameter, ranked in order of largest change in the ICER (the top 15 parameters are shown). In all cases, the ICER remained in quadrant 1 of the cost-effectiveness plane (Olara + Dox is more expensive and more effective than the comparator) indicated in the graph by a “1” at the end of each bar. AE = adverse event; Comp = comparator; Dox = doxorubicin; Gen Pop = general population; HR = hazard ratio; ICER = incremental cost-effectiveness ratio; LY = life year; Olara = olaratumab; Olara + Dox = olaratumab + doxorubicin; STS = soft tissue sarcoma; US = United States.

The one-way sensitivity analysis results are presented for the comparison of Olara + Dox with Dox in Figure 3; results for the other comparisons are presented in Supplementary Materials. The mean number of administrations of olaratumab was the parameter with the largest influence on the incremental cost per LY gained in the comparison of Olara + Dox with Dox, AIM, GemDoc (Maki), and PLD and was the second most-sensitive parameter in the comparison of Olara + Dox with GemDoc (GeDDiS) and MAID. None of the parameters considered in each pairwise comparison generated increases or reductions in the incremental cost per LY gained of more than $13,000 per LY gained. In the direct comparison with Dox, the highest ICER observed in the one-way sensitivity analysis was $114,717 per LY gained. In an additional analysis, drug costs for AIM, GemDoc (GeDDiS), GemDoc (Maki), PLD, and MAID were calculated assuming the same dose reduction as that observed for Dox in the JGDG study (rather than using the planned dose for these regimens as assumed in the base-case analysis). In pairwise comparisons, the ICER for Olara + Dox in this dose reduction scenario increased slightly compared with the base-case results (to $50,791, $78,692, $78,561, $101,831, and $65,217/LY gained versus AIM, GemDoc (GeDDiS), GemDoc (Maki), PLD, and MAID, resp.). In the scenario in which the cost of G-CSF was assumed to be zero, the ICER of Olara + Dox increased by nearly $19,000 and $16,000 per LY gained when compared with AIM and GemDoc (Maki), respectively.

### 3.3. Scenario Analyses

The scenario analyses explored the impact of alternative assumptions and parameter sources on ICERs. All other model settings were at base case, other than the parameter being investigated in the scenario analyses. A scenario exploring the impact of using PFS data as assessed by a blinded independent review showed a variation of less than 0.1% in the ICER of Olara + Dox versus Dox. Results for the direct comparison of Olara + Dox versus Dox ranged from $78,669 per LY gained to $171,593 per LY gained. The lowest ICER was associated with the scenario in which the treatment effect after trial follow-up was tapered to no treatment effect (HR = 1) over 4 years, starting at 32 months (the time of last observed death in the Olara + Dox arm of the JGDG trial) from the treatment effect observed in the JGDG study (HR = 0.463) [17]. The highest ICER was observed when the Weibull parametric function (proportional hazards) was used to estimate OS. The next two highest ICERs also were associated with alternative scenarios for OS, using alternative parametric survival functions with treatment arms modeled separately: log-normal ($121,725 per LY gained) and Gompertz ($165,839 per LY gained). The full range of scenarios exploring the direct comparison between Olara + Dox and Dox are presented in Supplementary Materials.

### 3.4. Probabilistic Sensitivity Analyses

**Figure 4:**
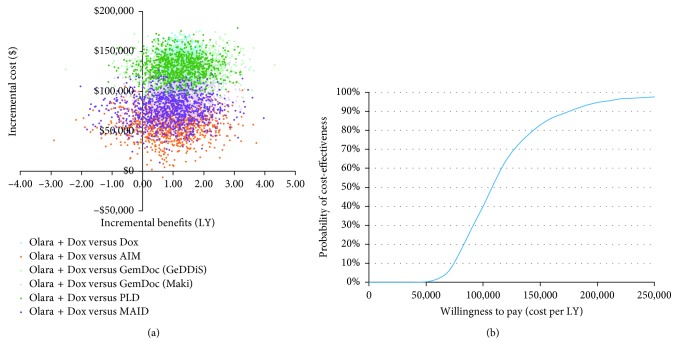
Probabilistic sensitivity analysis results. (a) Cost-effectiveness plane (base case); (b) cost-effectiveness acceptability curve (Olara + Dox versus Dox). (a) shows the joint distribution of incremental costs and LYs estimated by 1,000 model simulations for each treatment comparison in which the mean values for all model parameters were sampled simultaneously from their individual statistical distributions. Details of the distributions are presented in Supplementary Materials. (b) shows the probability of cost-effectiveness for Olara + Dox versus Dox as a function of the ICER threshold (the additional cost per LY gained that the decision-maker is willing to pay). From this and equivalent analyses for each pairwise treatment comparison, the probability that Olara + Dox is cost-effective at a willingness-to-pay threshold of $150,000 per LY gained was estimated as 83% versus Dox, 93% versus AIM, 93% versus GemDoc (GeDDiS), 94% versus GemDoc (Maki), 87% versus PLD, and 90% versus MAID. AIM = ifosfamide + doxorubicin + mesna; Dox = doxorubicin; GemDoc = gemcitabine + docetaxel; LY = life-year; MAID = mesna + doxorubicin + ifosfamide + dacarbazine; Olara + Dox = olaratumab + doxorubicin; PLD = pegylated liposomal doxorubicin.

Figure 4(a) shows the distributions of incremental costs and benefits (LYs gained) estimated by the probabilistic sensitivity analysis on the cost-effectiveness plane for Olara + Dox versus each comparator.  Figure 4(b) shows the cost-acceptability curve for the direct comparison between Olara + Dox and Dox, which describes the probability that Olara + Dox is expected to be cost-effective as a function of the willingness-to-pay threshold. In the probabilistic sensitivity analyses, Olara + Dox was always associated with higher costs and additional LYs than Dox but was dominated by other comparators in some probabilistic simulations. In the direct comparison with Dox, at a willingness-to-pay threshold of $50,000, $100,000, $150,000, and $200,000 per LY gained, the probability that Olara + Dox was cost-effective was 0%, 40%, 83%, and 95%, respectively. At a willingness-to-pay threshold of $150,000 per LY gained, the probability that Olara + Dox was cost-effective was 93%, 93%, 94%, 87%, and 90% when compared with AIM, GemDoc (GeDDiS), GemDoc (Maki), PLD, and MAID, respectively.

## 4. Discussion

This study evaluated the cost-effectiveness of Olara + Dox in the treatment of adult patients with anthracycline-naïve, advanced STS not amenable to curative treatment with radiotherapy or surgery.

The base-case results estimated that the ICER for Olara + Dox versus Dox was $105,408 per LY gained, and the probability of cost-effectiveness for Olara + Dox compared with Dox at willingness-to-pay thresholds of $50,000, $100,000, $150,000, and $200,000 per LY gained was 0%, 40%, 83%, and 95%, respectively. The mean total expected lifetime cost for patients receiving Olara + Dox was $60,818 to $133,653 higher than the comparators. Mean LYs were increased substantially for patients receiving Olara + Dox, by 1.20 to 1.68 LYs. In the fully incremental analysis, AIM, MAID, GemDoc (GeDDiS), GemDoc (Maki), and PLD were all dominated. Sensitivity and scenario analyses suggested that the results were most sensitive to uncertainty in the OS benefit for Olara + Dox.

The main strengths of the research were as follows. The model underwent extensive validation in accordance with best practices [25], and input data were identified by systematic reviews of clinical and economic evidence. The comparison of Olara + Dox versus Dox was based on data from a head-to-head, randomized, controlled trial (JGDG trial [17]) allowing comparison under same protocol with the same inclusion/exclusion criteria and the same clinical practice. PFS was estimated in the model using the JGDG trial's Kaplan–Meier data; OS was estimated using survival functions fitted to patient-level data from the trial, in line with accepted guidelines. The indirect comparisons of Olara+Dox against AIM and GemDoc (GeDDiS) were based on an NMA [23] using accepted Bayesian methods [41]. Administration of AIM was assumed to happen in an outpatient setting. This was a conservative assumption, as clinical expert opinion suggested it could also be administered in an inpatient setting which would suggest higher administration costs for AIM than those assumed in the model. If inpatient costs were to be applied in this model, AIM would have higher cost and therefore lesser value, which in turn may overestimate the value of other comparators versus this regimen. The use of the outpatient regimen ensured that the lowest cost option was included to provide the reader with the best possible outcome for AIM in the model. The model also used input data from a Surveillance, Epidemiology, and End Results-Medicare study [42]. Investigator-assessed PFS data, rather than blinded independent radiologically assessed PFS data, were used in the base-case because the former were expected to more closely reflect assessment of disease progression in routine clinical practice. A scenario analysis exploring the impact of using blinded independent radiologically assessed data indicated that the ICER was not sensitive to the PFS data used. OS estimates for Dox at 5 to 10 years predicted by the model (using the gamma function in the base-case analysis) were consistent with observed long-term survival for a similar population (specifically, a large meta-analysis of trials investigating anthracycline-containing first-line therapy reported by van Glabbeke et al. [9]). Owing to the uncertainty in the OS prediction beyond the end of the JGDG trial follow-up, a range of alternative methods for OS prediction were explored. The base-case analysis made a conservative assumption that there is no continuation of treatment effect for Olara + Dox beyond the last mortality event observed in the Olara + Dox arm of the JGDG trial (32 months).

Extensive sensitivity analyses were performed using the economic model, including univariate and probabilistic sensitivity analyses incorporating all model parameters and scenario analyses exploring structural uncertainty. In addition, a range of analyses were performed to explore the potential for bias in the JGDG trial OS data resulting from the use of single-agent olaratumab after disease progression in the Dox arm. All these analyses supported the study conclusions related to the effect of Olara + Dox on OS.

Several limitations of the research should be noted. The efficacy data for Olara + Dox used in the direct comparison were based on a phase 2 trial with a proportion of patients surviving at the end of the follow-up period (27 (40.9%) in the Olara + Dox arm and 15 (22.4%) in the Dox arm) [17]. This and the fact that the study's longest follow-up was under 4 years, whereas cost-effectiveness models require a lifetime horizon in order to capture all potential costs (25 years), led to substantial uncertainty in the economic model estimate for the long-term OS, particularly for Olara + Dox (since the model predictions for Olara + Dox could not be validated as they were for Dox using longer-term OS data). Results from the ongoing phase 3 trial (estimated completion in 2020 (ANNOUNCE, NCT02451943; Eli Lilly and Company [18])) will provide additional data and help to reduce some uncertainty in the economic model. We recommend that further evaluation of the cost-effectiveness of Olara + Dox is performed when the phase 3 trial data are available to incorporate the additional evidence related to treatment costs, safety, and the benefits of treatment into the economic model.

The indirect comparisons had several limitations: First, trials for AIM and GemDoc (GeDDiS) investigated first-line therapy only; in order to provide analyses in any line of therapy (as was investigated in the JGDG trial), the efficacy of these regimens was assumed to be independent of the line of therapy. Second, the indirect comparison was performed by combining parametric survival functions fitted to the JGDG trial data, with HRs originally estimated from the Judson et al. [12] and the GeDDiS [19] trials using other methods and synthesized using NMA. Although this is a commonly accepted approach in economic models in oncology, its limitations should be recognized [23, 34]. Third, it was not possible to use conventionally accepted methods to perform indirect comparisons of the efficacy of Olara + Dox versus PLD, GemDoc (Maki), and MAID. These drugs could not be connected to the evidence network via randomized, controlled trials. Therefore, an assumption had to be made regarding their relative efficacy; specifically, PLD and GemDoc (Maki) were assumed to have the same efficacy as Dox and MAID was assumed to have the same efficacy as AIM. Lastly, to estimate drug and administration costs for Olara + Dox and Dox, we accounted for dose reductions and dose delays by using the mean dose and mean number of administrations observed in the JGDG trial. However, for the other comparators, insufficient information was available to estimate mean doses; therefore, the planned doses were used (a scenario analysis explored the impact of assuming dose reductions equivalent to that observed for Dox in the JGDG study; the ICER for Olara + Dox increased by less than $100 per LY gained in each pairwise comparison).

In the indirect comparisons with AIM and GemDoc (Maki), prophylactic G-CSF was assumed to be given using pegfilgrastim. The scenario analysis, assuming that the cheaper filgrastim is used (which assumed the cost of G-CSF to be zero), produced higher ICERs for the pairwise comparisons of Olara + Dox with AIM and GemDoc (Maki). However, in the fully incremental analysis these regimens were still dominated or extendedly dominated.

Further limitations regarding the health-state utility estimates used in the secondary analysis (which estimated the incremental cost per QALY gained) should be noted. First, data reported by Reichardt et al. [43] were based on a small sample (the sample size for each health state ranged from 12 to 35 patients); patients in the Reichardt study could have bone sarcoma and were required to have had a response to at least one line of treatment. These criteria differed from the population indicated for Olara + Dox. Second, the data used for the postprogression health state after second- or subsequent-line therapy extracted from a study by Delea et al. [44] were of poor quality because the authors used estimates from two separate studies (one combined EQ-5D trial data and European Organisation for Research and Treatment of Cancer Quality of Life Questionnaire trial data mapped to the EQ-5D, and the second was based on a health-state description (vignette) for progressive disease), and the methods used to derive the final estimate in the Delea et al. [44] study were unclear.

## 5. Conclusions

From a US payer perspective, the results of the base-case fully incremental analysis suggested that all interventions were expected to be dominated or extendedly dominated by Dox or Olara + Dox. The direct comparison of Olara + Dox with Dox resulted in an ICER of $105,408 per LY gained. The probability that Olara + Dox is cost-effective at willingness-to-pay thresholds of $50,000, $100,000, $150,000, and $200,000 per LY gained was 0%, 40%, 83%, and 95%, respectively. One-way sensitivity analyses showed that the mean number of Olara administrations in the Olara + Dox arm was the parameter with the largest influence on the ICER per LY gained, while scenario analyses suggested that uncertainty in the OS estimates was most influential overall. A range of scenarios resulted in ICERs varying from $78,669 to $171,593 per LY gained. Uncertainty in the economic model is expected to be reduced when the results of the ongoing phase 3, randomized, controlled trial (ANNOUNCE, NCT02451943) become available.

## Figures and Tables

**Table 1:** Study interventions.

Regimen	Drug	Planned (mean) dose per treatment cycle^a^	Duration of treatment (mean administrations)	Route	Clinical trial	PFS and OS data in the model
Olara + Dox	Olara	15 (14.0) mg/kg on day 1 and day 8	21-day treatment cycles until disease progression (19.4 administrations)^b^	IV infusion (60 minutes)	Tap et al. [17]	PFS: JGDG KM curve OS: gamma function, mean parameters (SE)^c^ Intercept: 2.38 (0.34) Scale: 1.08 (0.08) Treatment—Dox: −0.66 (0.43) LOT, first line: 0.56 (0.29) Treatment × LOT: −0.34 (0.44) Tumor—LMS: −0.29 (0.34) Treatment × tumor: 0.40 (0.48) Sex—male: −0.48 (0.21) ECOG score of 0 : 0.91 (0.22) Shape: −0.68 (0.40)
	Dox	75 (73.7) mg/m^2^ on day 1	Up to eight 21-day treatment cycles or disease progression (5.7 administrations)	IV push		
	Dex	750 (707.0) mg/m^2^ on day 1	Treatment cycles 5–8 (3.6 administrations for 59% of patients)	IV infusion within 30 minutes prior to every Dox infusion		
Dox	Dox	75 (74.7) mg/m^2^ on day 1	Up to eight 21-day treatment cycles or until disease progression (4.4 administrations)	IV push (15–60 minutes)		
	Dex	750 (725.8) mg/m^2^ on day 1	Treatment cycles 5–8 (3.1 administrations for 45% of patients)	IV infusion within 30 minutes prior to every Dox infusion		

AIM	Ifo	2.5 g/m^2^ on day 1, day 2, day 3, and day 4	Up to six 21-day treatment cycles or until disease progression (4.4 cycles)^d^	IV infusion (60 minutes)	Judson et al. [12]	HR (95% CrI)^e^ Olara + Dox versus AIM PFS: 0.907 (0.573, 1.438)^f^ OS: 0.559 (0.343, 0.897) NMA [23]
	Dox	25 mg/m^2^ on day 1, day 2, and day 3		IV push		
	Mesna	2 g/m^2^ on day 1, day 2, day 3, and day 4	Each cycle	IV infusion and IV push		
	G-CSF	6 mg pegfilgrastim on day 5	Each cycle	SC		
	Dex^i^	250 mg/m^2^,^g^ on day 1, day 2, and day 3	Treatment cycles 5 and 6	IV infusion		

GemDoc (GeDDiS)	Gem	675 mg/m^2^ on day 1 and day 8	Up to six 21-day treatment cycles or until disease progression (4.1 cycles)^h^	IV infusion (90 minutes)	Seddon et al. [19]	HR (95% CrI)^e^ Olara + Dox versus GemDoc PFS: 0.522 (0.321, 0.862)^f^ OS: 0.432 (0.252, 0.744) NMA [23]
	Doc	75 mg/m^2^ on day 8		IV infusion (60 minutes)		

GemDoc (Maki)	Gem	900 mg/m^2^ on day 1 and day 8	Up to six or eight 21-day treatment cycles or until disease progression (4.0 cycles)^j^	IV infusion (90 minutes)	Maki et al. [21]	Efficacy is assumed to be equivalent to Dox in the JGDG trial
	Doc	100 mg/m^2^ on day 8		IV infusion (60 minutes)		
	G-CSF	6 mg pegfilgrastim on day 9		SC		

PLD	PLD	50 mg/m^2^ on day 1	Up to six 28-day treatment cycles or until disease progression (3.4 cycles)	IV infusion (60 minutes)	Judson et al. [22]	Efficacy is assumed to be equivalent to Dox in the JGDG trial^k^

MAID	Mesna	2.5 g/m^2^ on day 1, day 2, and day 3	Up to six 21-day treatment cycles or until disease progression (4.4 cycles)^l^	IV infusion (60 minutes)	Bui-Nguyen et al. [20]	Efficacy is assumed to be equivalent to AIM
	Dox	20 mg/m^2^ on day 1, day 2, and day 3		IV infusion (<60 minutes or IV push)		
	Ifo^m^	2.5 g/m^2^ on day 1, day 2, and day 3		IV infusion (180 minutes)		
	DTIC	300 mg/m^2^ on day 1, day 2, and day 3		IV infusion (60 minutes)		
	Dex^i^	200 mg/m^2^,^g^ on day 1, day 2, and day 3	Treatment cycles 5 and 6	IV infusion		

AIM = ifosfamide + doxorubicin + mesna; CrI = credible interval; Dex = dexrazoxane; Dox = doxorubicin; DTIC = dacarbazine; ECOG = Eastern Cooperative Oncology Group; G-CSF = granulocyte-colony stimulating factor; Gem = gemcitabine; GemDoc = gemcitabine + docetaxel; HR = hazard ratio; Ifo = ifosfamide; ITT = intention-to-treat; IV = intravenous; KM = Kaplan–Meier; LMS = leiomyosarcoma; LOT = line of treatment; MAID = mesna + doxorubicin + ifosfamide + dacarbazine; NMA = network meta-analysis; Olara = olaratumab; Olara+Dox = olaratumab + doxorubicin; OS = overall survival; PFS = progression-free survival; PLD = pegylated liposomal doxorubicin; SC = subcutaneous; SE = standard error. ^a^Mean dose was available only for Olara + Dox and Dox from the JGDG trial. For the other regimens, the planned dose was assumed. ^b^In the JGDG trial, all patients had discontinued their randomized treatment at the data cutoff point; therefore, there was no need for prediction of treatment costs beyond trial follow-up. ^c^The survival function for Olara + Dox assumed no treatment effect after 32 months (i.e., applied a HR of 1.00). Both functions were adjusted to account for increased risk of death from other causes. ^d^Estimated from Judson et al. [24]. ^e^Estimated using stratified ITT analysis HR for Olara + Dox versus Dox in the NMA. ^f^Estimated using the investigator-assessed PFS HR for Olara + Dox versus Dox in the NMA. ^g^Ten times the Dox dose [15]. ^h^Assumed to equal to that for Dox in the trial by Judson et al. [12]; estimated from Judson et al. [24]. ^i^Dex was not administered with AIM and MAID in the studies by Judson et al. [12] and Bui-Nguyen et al. [20], respectively. However, as Dex was administered to patients receiving Dox from cycles 5 to 8 in the JGDG trial, it was assumed that a proportion of patients receiving five and six cycles of AIM or MAID also received Dex. ^j^Based on the median number of cycles reported by Maki et al. [21]. ^k^Judson et al. [22] reported equivalent antitumor activity for Dox and PLD. ^l^Assumed the same as AIM. ^m^Normal saline (1000 mL) is administered for 2 hours after each Ifo dose. The associated administration cost was added to the Ifo administration cost; the cost of the normal saline product was assumed to be negligible.

**Table 2:** Key input parameters.

Drug prices (source: Micromedex Solutions [30])^a^			
Drug	Vial size and price per vial	Vial size and price per vial	Minimum price per milligram
Olara	190 mg vial $896.80	500 mg vial $2,360.00	$4.72
Dox	20 mg vial $5.44	50 mg vial $13.60	$0.84
Dex	250 mg vial $163.46	500 mg vial $326.91	$1.10
Ifo	1000 mg vial $29.78	3000 mg vial $89.34	$0.05
Mesna	1000 mg vial $15.25	—	$0.07
G-CSF	6 mg vial $3,898.41	—	$859.28
Gem	200 mg vial $8.09	1000 mg vial $40.44	$0.09
Doc	20 mg vial $47.76	80 mg vial $191.04	$8.42
PLD	20 mg vial $969.00	50 mg vial $2,422.50	$48.45
DTIC	100 mg vial $4.36	200 mg vial $8.72	$0.10

Administration costs (per administration)			
(source: HCPCS codes from essential RBRVS [31])			

Drug	Administration day and cost	Administration day and cost	Administration day and cost
Olara + Dox	Day 1 of cycles 1–4 (Olara + Dox) $379.76	Day 1 of cycles 5–8 (Olara + Dox + Dex) $476.21	Day 8 of all cycles (Olara only)^b^ $208.79
Dox	Day 1 of cycles 1–4 (Dox) $170.98	Day 1 of cycles 5–8 (Dox + Dex) $379.76	—
AIM^c^	Days 1, 2, and 3 of cycles 1–4 (AIM) $572.11	Days 1, 2, and 3 of cycles 5–6 (AIM + Dex) $668.56	Day 4 (Ifo + mesna only) $476.21
GemDoc	Day 1 (Gem) $252.63	Day 8 (GemDoc) $349.08	—
PLD	Day 1 (PLD) $208.79	—	—
MAID	Days 1, 2, and 3 (MAID) $772.13	Days 1, 2, and 3 (MAID + Dex) $868.58	—
G-CSF	Days 5 and 9 $115.08	—	—

Monitoring			
(sources: clinical expert opinion; HCPCS codes from essential RBRVS [31])			

Resource category	Cost per visit and tests	Frequency of visits	Cost per resource use
Follow-up visit	Cost per visit $723.79^d^	Every 3 months for 5 years, every 6 months until 7 years, annually thereafter^e^	—
Cardiac monitoring	Multigated acquisition scan	Every Dox cycle^e^	$366.06^f^
Cardiac monitoring	Echocardiography	Every second Dox cycle^e^	$352.36^f^

Total cost of all subsequent lines of active systemic therapy			
(sources: JGDG trial in Lilly data on file [32]; HCPCS codes from essential RBRVS [31]^g^)			

Drug	Drug cost	Administration cost	AE costs
Olara + Dox patients	$5,418.43	$3,972.43	$21,303.98
Patients receiving comparator interventions	$5,515.48	$4,043.58	$21,303.98

AE = adverse event; AIM = ifosfamide + doxorubicin + mesna; BSA = body surface area; Dex = dexamethasone; Doc = docetaxel; Dox = doxorubicin; DTIC = dacarbazine; G-CSF = granulocyte-colony stimulating factor; Gem = gemcitabine; GemDoc = gemcitabine + docetaxel; HCPCS = Healthcare Common Procedure Coding System; Ifo = ifosfamide; MAID = mesna + doxorubicin + ifosfamide + dacarbazine; Olara = olaratumab; Olara+Dox = olaratumab + doxorubicin; PLD = pegylated liposomal doxorubicin; RBRVS = resource-based relative value scale; SD = standard deviation; UK = United Kingdom. ^a^The mean (SD) weight and BSA were assumed to be 85.8 (23.0) kg and 2.0 (0.3) m^2^ (JGDG trial, Lilly data on file [33]). Drug costs were calculated by assuming that unused drug in opened vials is wasted. The distribution of weight and BSA was simulated, and the number of vials needed for each weight per BSA category was determined and costed. ^b^Also day 1 after Dox discontinuation (Olara only). ^c^Assumed to be given in an outpatient setting. ^d^Outpatient visit and physical examination ($223.03); computerized tomography scan ($352.78; 92% of visits); positron emission tomography ($1,072.98; 9% of visits); magnetic resonance imaging ($584.29; 14% of visits). Source of unit costs: Essential RBRVS [31] HCPCS codes; usage of imaging based on Lilly observational study [32] (UK data). ^e^Based on clinical expert opinion. ^f^Essential RBRVS [31] HCPCS codes. ^g^Costs were estimated based on subsequent lines of treatment observed after the investigational therapy in the JGDG study in each treatment arm. Subsequent therapies included DTIC, GemDoc, Dox, eribulin, everolimus, Gem, Ifo, Ifo + mesna, pazopanib, and trabectedin. The average cost of treatments in the Olara + Dox arm was applied for both arms. The total cost of all subsequent treatments recorded in the trial was estimated based on the proportion of patients receiving each regimen and duration of therapy recorded. Drug and administration costs were estimated assuming a 3-week treatment cycle and dosing schedules from clinical studies; unit costs were from Micromedex Solutions [30] and HCPCS codes from Essential RBRVS [31].

**Table 3:** Economic analysis results.

	Olara + Dox	AIM	MAID	GemDoc (Maki)	PLD	GemDoc (GeDDiS)	Dox
*Base-case results*							
Median PFS^a^ (months)	6.5	6.0	6.0	3.9	3.9	2.8	3.9
Median OS^a^ (months)	24.0	14.3	14.3	11.5	11.5	12.0	11.5
10-year survival (%)	9.5	4.7	4.7	5.0	5.0	2.9	5.0
Mean LYs (95% CrI)	3.37 (2.53–4.36)	2.17 (1.24–3.44)	2.17 (1.26–3.35)	2.10 (1.55–2.96)	2.10 (1.53–2.98)	1.69 (0.91–2.85)	2.10 (1.55–2.94)
Mean total expected lifetime cost ($) (per patient (95% CrI))	182,984 (157,673–207,429)	122,166 (106,753–138,696)	104,787 (90,428–119,603)	83,473 (69,478–99,681)	53,925 (44,335–65,606)	50,976 (43,686–59,751)	49,330 (43,058–58,480)
*Incremental cost ($) (per LY* ^b^)							
Each comparator versus Dox	105,408	1,064,437	810,457	Dominated	Dominated	Dominated	Reference
Fully incremental, each comparator versus previous nondominated alternative	105,408	Dominated	Extendedly dominated	Dominated	Dominated	Dominated	NA
Pairwise, Olara + Dox versus each comparator (per LY)	—	50,701	65,189	78,480	101,784	78,679	105,408
*Secondary analysis*							
Mean QALYs per patient (95% CrI)	1.86 (1.40–2.42)	1.21 (0.73–1.89)	1.21 (0.74–1.83)	1.17 (0.88–1.63)	1.17 (0.87–1.64)	0.94 (0.54–1.57)	1.17 (0.87–1.61)
*Incremental cost ($) (per QALY)*							
Each comparator versus Dox	196,309	1,841,157	1,642,925	Dominated	Dominated	Dominated	Reference
Fully incremental, each comparator versus previous nondominated alternative	196,309	Extendedly dominated	Extendedly dominated	Dominated	Dominated	Dominated	NA
Pairwise, Olara + Dox versus each comparator (per QALY)	—	94,839	120,846	145,447	189,147	143,732	196,309

AIM = ifosfamide + doxorubicin + mesna; CrI = credible interval (within which 95% of probabilistic simulations were observed); Dox = doxorubicin; GemDoc = gemcitabine + docetaxel; ICER = incremental cost-effectiveness ratio; LY = life-year; MAID = mesna + doxorubicin + ifosfamide + dacarbazine; NA = not applicable; Olara + Dox = olaratumab + doxorubicin; OS = overall survival; PFS = progression-free survival; PLD = pegylated liposomal doxorubicin; QALY = quality-adjusted life year. Cost and outcomes (with the exception of median PFS, OS and 10-year survival) are discounted at 3%. ^a^Calculated from the survival functions used in the model. ^b^Dominated: a dominated intervention is defined as an intervention with higher costs and worse outcomes than an alternative intervention. Extendedly dominated: in a fully incremental analysis, a treatment is said to be extendedly dominated when the treatment's ICER is higher than the ICER of the next, more effective, alternative (i.e., the given treatment is dominated by the combination of two alternatives and should not be used to calculate appropriate ICERs). For example, consider that there are three drug regimens, A, B, and C, with regimen C being more effective (resulting in greater LYs) and more costly than regimen B and regimen B being more effective (resulting in greater LYs) and more costly than regimen A. Drug regimen C is said to extendedly dominate drug regimen B if the ICER for drug regimen C when compared with drug regimen A is more favorable (has a lower value) than the ICER for drug regimen B when compared with drug regimen A.

## Supplementary Materials

Supplementary Materials Table S-1: key design features of the systematic review of economic studies. Figure S-1: PRISMA diagram for the systematic review of economic studies. Table S-2: studies identified in the systematic review of economic studies. Figure S-2: economic model structure and health-utility estimates. Table S-3: summary of base-case incidence of adverse events applied in the economic model. Table S-4: summary of mean number of adverse events per patient having the event. Table S-5: summary of base-case grade ≥ 3 adverse-event costs and utility decrements applied in the economic model. Table S-6: summary of base-case grade 1-2 adverse-event costs and utility decrements applied in the economic model. Table S-7: summary of base-case variables applied in the economic model. Figure S-2: tornado diagram for Olara + Dox versus AIM: change in ICER (US $ per life-year saved). Figure S-3: tornado diagram for Olara + Dox versus GemDoc (GeDDiS): change in ICER (US $ per LY saved). Figure S-4: tornado diagram for Olara + Dox versus GemDoc (Maki): change in ICER (US $ per LY saved). Figure S-5: tornado diagram for Olara + Dox versus PLD: change in ICER (US $ per LY saved). Figure S-6: tornado diagram for Olara + Dox versus MAID: change in ICER (US $ per LY saved). Table S-8: scenario analysis results: direct comparison of Olara + Dox with Dox. Table S-9: scenario analysis results: indirect comparison of Olara + Dox with AIM, GemDoc (GeDDiS), GemDoc (Maki), PLD, and MAID.
