# Supplementary material for: Cost-Effectiveness of Olaratumab in Combination with Doxorubicin for Patients with Soft Tissue Sarcoma in the United States
Source: Sarcoma. 2018 Mar 26;2018:6703963. doi: 10.1155/2018/6703963 (PMC5892240; doi:10.1155/2018/6703963)
Supplement: Supplementary Materials — Table S-1: key design features of the systematic review of economic studies. Figure S-1: PRISMA diagram for the systematic review of economic studies. Table S-2: studies identified in the systematic review of economic studies. Figure S-2: economic model structure and health-utility estimates. Table S-3: summary of base-case incidence of adverse events applied in the economic model. Table S-4: summary of mean number of adverse events per patient having the event. Table S-5: summary of base-case grade ≥ 3 adverse-event costs and utility decrements applied in the economic model. Table S-6: summary of base-case grade 1-2 adverse-event costs and utility decrements applied in the economic model. Table S-7: summary of base-case variables applied in the economic model. Figure S-2: tornado diagram for Olara + Dox versus AIM: change in ICER (US $ per life-year saved). Figure S-3: tornado diagram for Olara + Dox versus GemDoc (GeDDiS): change in ICER (US $ per LY saved). Figure S-4: tornado diagram for Olara + Dox versus GemDoc (Maki): change in ICER (US $ per LY saved). Figure S-5: tornado diagram for Olara + Dox versus PLD: change in ICER (US $ per LY saved). Figure S-6: tornado diagram for Olara + Dox versus MAID: change in ICER (US $ per LY saved). Table S-8: scenario analysis results: direct comparison of Olara + Dox with Dox. Table S-9: scenario analysis results: indirect comparison of Olara + Dox with AIM, GemDoc (GeDDiS), GemDoc (Maki), PLD, and MAID. [file 6703963.f1.pdf]

ONLINE SUPPLEMENT

Table S-1. Key Design Features of the Systematic Review of Economic Studies

Feature	Economic Review Design
Databases	▪ MEDLINE, MEDLINE In-Process (PubMed platform)<br>▪ Embase (Elsevier platform)<br>▪ BIOSIS<br>▪ EconLit<br>▪ Cochrane Library (including the NHS Economic Evaluation Database and the Health Technology Assessment database)
Other Searches	▪ Bibliographies of relevant robust systematic literature reviews, economic analyses, and health technology assessments<br>▪ Health technology assessment websites (NICE; Scottish Medicines Consortium; All Wales Medicines Strategy Group; The Canadian Agency for Drugs and Technologies in Health; The International Network of Agencies for Health Technology Assessment)
Date range	▪ 1 January 2004 to 2 January 2015; review update extended to 26 September 2016
Language	▪ No language restrictions
Screening	▪ Independent double-screening of titles and/or abstracts (level 1) and then full texts (level 2) using prespecified inclusion and exclusion criteria in PICOS framework, with reconciliation of discrepancies<br>▪ Reasons for exclusion recorded

Feature	Economic Review Design
Population	▪ Adult patients with advanced or metastatic STS not amenable for surgery or radiotherapy (all histologies)
Interventions and comparators	▪ All pharmacological treatments for advanced or metastatic STS in any therapy line
Outcomes	▪ No criteria for reported outcomes were specified
Study design	▪ Economic evaluations (cost-effectiveness, cost-minimization, cost-benefit , and cost-utility analyses)<br>▪ Retrospective studies reporting costs or resource utilization (e.g., cost-of-illness and cross-sectional studies)<br>▪ Prospective studies reporting utilities, costs, or resource utilization (e.g., observational studies and clinical trials)<br>▪ Utility studies (including studies where utility weights were mapped from other instruments, e.g., disease-specific patient-reported outcome measures)
Data extraction	▪ Single extraction performed by one researcher, with a quality-check of all data by a second researcher not involved with the extraction
Quality assessment of included studies	▪ Quality assessment of economic evaluations using the checklist recommended by NICE [1] (based on Drummond and Jefferson [2])<br>▪ Resource use and cost estimates were evaluated for relevance to the economic model<br>▪ Utility estimates were evaluated for compliance with the NICE reference case

BIOSIS = Biological Information Services; NHS = National Health Service; NICE = National Institute for Health and Care Excellence; PICOS = populations, interventions, comparators, outcomes, and study design; STS = soft tissue sarcoma.

Figure S-1. PRISMA Diagram for the Systematic Review of Economic Studies

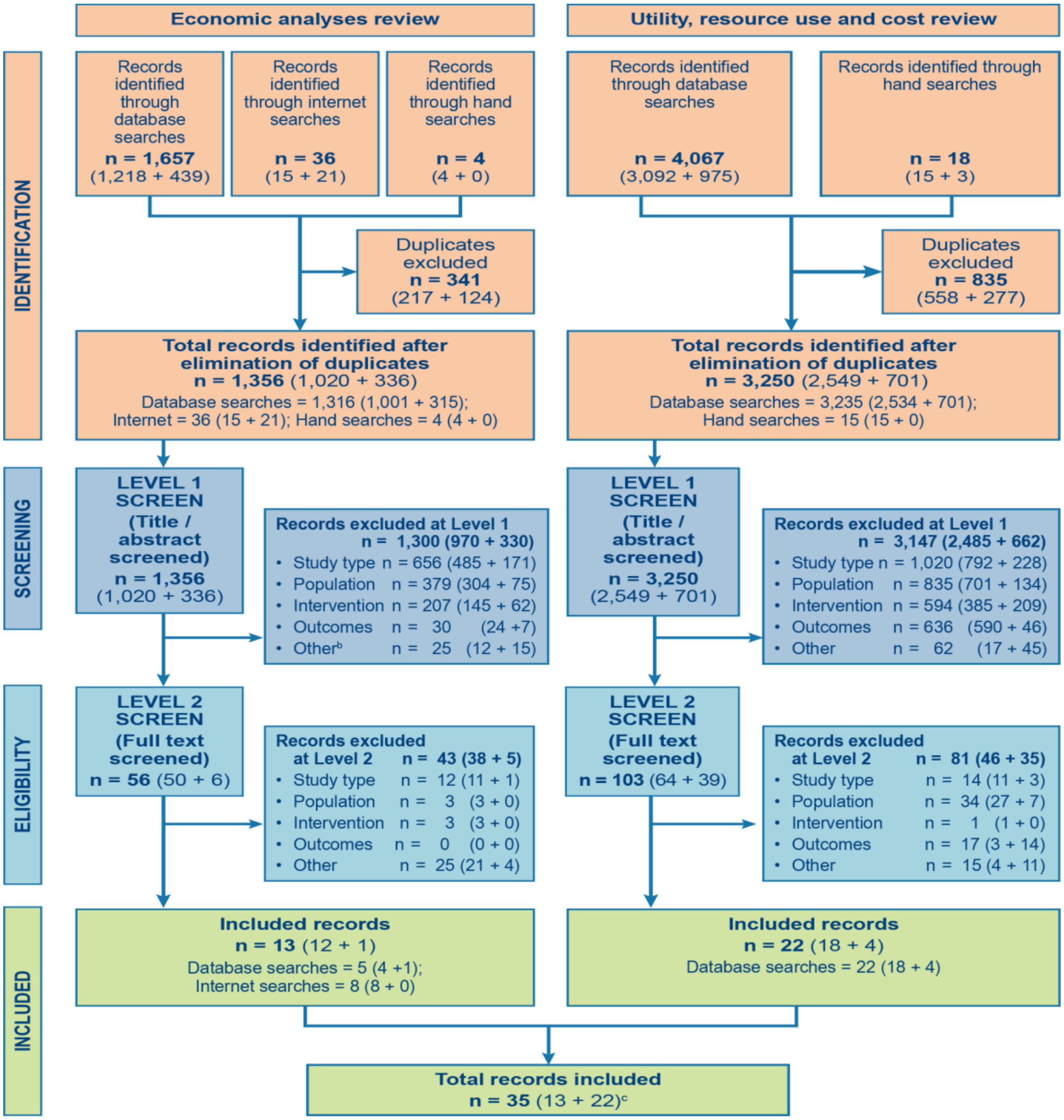

PRISMA = Preferred Reporting Items for Systematic Reviews and Meta-Analyses.

[a] Numbers in the diagram represent the total number of records identified (original review + update)

[b] Reasons categorized as “other” include duplicate articles; articles that have been withdrawn from publication; and conference abstracts published before January 2012 and January 2015 in the original and update review, respectively.

[C] “Total records included” represent combined totals (combined original and update totals in the economic analyses review + combined original and update totals in the cost, resource-use, and utility review). Of these, 30 (12 economic evaluations and 18 cost, resource-use, and utility studies) were identified in the original review and 5 (1 economic evaluation and 4 cost, resource-use, and utility studies) were identified in the update review.

Note: Some of the 35 records included in the systematic review had more than one type of data, e.g., economic evaluations and cost, resource-use, and utility estimates.

Table S-2. Studies Identified in the Systematic Review of Economic Studies

1. All Wales Medicines Strategy Group (AWMSG). Trabectedin (Yondelis). Reference No. 318. September 2008. Available at: http://www.awmsg.org/awmsgonline/grabber;jsessionid=40334b58b6d4ebfba7dd8b449d0b?resId=394. Accessed 8 February 2015.
2. All Wales Medicines Strategy Group (AWMSG). Pazopanib (Votrient). Reference No. 549. December 2013. Available at: http://www.awmsg.org/awmsgonline/grabber?resId=1148. Accessed 8 February 2015.
3. Amdahl J, Manson SC, Isbell R, Chit A, Delea TE. Utility mapping of the EORTC QLQ-C30 onto EQ-5D in patients with soft tissue sarcoma. Value Health. 2013;16(7):A419.
4. Amdahl J, Manson SC, Isbell R, Chit A, Diaz J, Lewis L, et al. Cost-effectiveness of pazopanib in advanced soft tissue sarcoma in the United Kingdom. Sarcoma. 2014;2014:481071. doi: 10.1155/2014/481071.
5. Amdahl J, Manson S, Isbell R, Chit AN, Diaz JR, Lewis L, et al. Cost effectiveness of pazopanib in soft tissue sarcoma. Value Health. 2012;15(7):A423-4.
6. Canadian Agency for Drugs and Technologies in Health. Votrient for soft tissue sarcoma. July 2012. Available at: https://www.cadth.ca/sites/default/files/pcodr/pcodr-votrientsts-fn-rec.pdf. Accessed 8 February 2015.
7. Conter HJ. Financial risk-sharing agreements: using options to make marginal benefits cost effective. J Clin Oncol. 2016;34(7).
8. Coriat R, Mir O, Camps S, Ropert S, Billemont B, Leconte M, et al. Ambulatory administration of 5-day infusion ifosfamide+mesna: a pilot study in sarcoma patients. Cancer Chemother Pharmacol. 2010 Feb;65(3):491-5.
9. Davidson D, Barr RD, Riad S, Griffin AM, Chung PW, Catton CN, et al. Health-related quality of life following treatment for extremity soft tissue sarcoma. J Surg Oncol. 2016 Dec;114(7):821-7.
10. Delea TE, Amdahl J, Nakhaipour HR, Manson SC, Wang A, Fedor N, et al. Cost-effectiveness of pazopanib in advanced soft-tissue sarcoma in Canada. Curr Oncol. 2014 Dec;21(6):e748-59.
11. Duh MS, Hackshaw MD, Ivanova JI, Kruse G, Miller LA, Lefebvre P, et al. Costs associated with intravenous cancer therapy administration in patients with metastatic soft tissue sarcoma in a US population. Sarcoma. 2013;2013:947413.
12. Engel-Nitz NM, Song R, Horstman TV. Ifosfamide treatment of patients with soft tissue sarcoma: health care

utilization and cost implications. Value Health. 2013;16(3):A134.

13. Gelderblom H, Blay JY, D'Adamo D, Hudgens S, Kontoudis I, Le Cesne A, et al. Randomized, open-label, multicenter, phase 3 study of eribulin versus dacarbazine in patients with leiomyosarcoma and adipocytic sarcoma: health-related quality of life results. Eur J Cancer. 2015;51:S702.

14. Gerrand CH, Billingham LJ, Woll PJ, Grimer RJ. Follow up after primary treatment of soft tissue sarcoma: a survey of current practice in the United Kingdom. Sarcoma. 2007;2007:34128.

15. Guest JF, Panca M, Sladkevicius E, Gough N, Linch M. Cost effectiveness of first-line treatment with doxorubicin/ifosfamide compared to trabectedin monotherapy in the management of advanced soft tissue sarcoma in Italy, Spain, and Sweden. Sarcoma. 2013a;2013:725305.

16. Guest JF, Sladkevicius E, Gough N, Linch M, Grimer R. Utility values for advanced soft tissue sarcoma health states from the general public in the United Kingdom. Sarcoma. 2013b;2013:863056.

17. Gundle KR, Cizik AM, Punt SE, Conrad EU III, Davidson DJ. Validation of the SF-6D health state utilities measure in lower extremity sarcoma. Sarcoma. 2014;2014:450902.

18. Haupais H, Caussin M, Basuyau F, Guillemet C, Doucet J, Remy E. Trabectedin for treatment of advanced soft-tissue sarcoma: a 18 months regional evaluation. Int J Clin Pharm. 2012;34(1):222.

19. Jönsson L, Justo N, Musayev A, Krishna A, Burke T, Pellissier J, et al. Cost of treatment in patients with metastatic soft tissue sarcoma who respond favourably to chemotherapy. The Sarcoma Treatment and Burden of Illness in North America and Europe (SABINE) study. Eur J Cancer Care (Engl). 2016 May;25(3):466-77.

20. Kozma CM, Slaton TL, McKenzie RS. Healthcare resource utilization and cost considerations in patients with soft tissue sarcoma treated with chemotherapy. J Clin Oncol. 2015;33(15).

21. Leahy M, Reichardt P, García Del Muro X, Pisters P, Chawla S, Martín J, et al. Health-related quality of life in patients with metastatic sarcoma—the Sarcoma Treatment and Burden of Illness Study in North America and Europe (Sabine) Study. Ann Oncol. 2010;21.

22. Liniker E, Harrison M, Weaver JMJ, Agrawal N, Chhabra A, Kingshott V, et al. Treatment costs associated with interventional cancer clinical trials conducted at a single UK institution over 2 years (2009-2010). Br J Cancer. 2013;109(8):2051-7.

23. National Institute for Health and Care Excellence. Trabectedin for the treatment of advanced soft tissue sarcoma. Technology appraisal guidance 185. 2010. Available at: http://www.nice.org.uk/guidance/ta185/resources/guidance-trabectedin-for-the-treatment-of-advanced-soft-

tissue-sarcoma-pdf. Accessed 8 February 2015.

24. Perrier L, Buja A, Mastrangelo G, Vecchiato A, Sandona P, Ducimetiere F, et al. Clinicians' adherence versus non adherence to practice guidelines in the management of patients with sarcoma: a cost-effectiveness assessment in two European regions. BMC Health Serv Res. 2012;12:82.

25. Porter GA, Cantor SB, Walsh GL, Rusch VW, Leung DH, DeJesus AY, et al. Cost-effectiveness of pulmonary resection and systemic chemotherapy in the management of metastatic soft tissue sarcoma: a combined analysis from the University of Texas M.D. Anderson and Memorial Sloan-Kettering Cancer Centers. J Thorac Cardiovasc Surg. 2004 May;127(5):1366-72.

26. Rafia R, Simpson E, Stevenson M, Papaioannou D. Trabectedin for the treatment of advanced metastatic soft tissue sarcoma: a NICE single technology appraisal. Pharmacoeconomics. 2013 Jun;31(6):471-8.

27. Reichardt P, Leahy M, García del Muro X, Ferrari S, Martín J, Gelderblom H, et al. Quality of life and utility in patients with metastatic soft tissue and bone sarcoma: the Sarcoma Treatment and Burden of Illness in North America and Europe (SABINE) study. Sarcoma. 2012;2012:740279.

28. Scottish Medicines Consortium. Advice: pazopanib (Votrient). SMC No. 820/12. December 2012. Available at: http://www.scottishmedicines.org.uk/files/advice/pazopanib_Votrient_FINAL_November_2012_Amended_051212_for_website.pdf. Accessed 8 February 2015.

29. Scottish Medicines Consortium. Advice: trabectedin (Yondelis). SMC No. 452/08. August 2008. First submission. Available at: http://www.scottishmedicines.org.uk/files/trabectedin__Yondelis__FINAL_July_2008.doc_for_website.pdf. Accessed 8 February 2015.

30. Scottish Medicines Consortium. Advice: trabectedin (Yondelis). SMC No. 452/08. November 2010. Resubmission. Available at: http://www.scottishmedicines.org.uk/files/advice/trabectedin_Yondelis_RESUBMISSION_FINAL_October_2010.doc_for_website.pdf. Accessed 8 February 2015.

31. Scottish Medicines Consortium. Advice: trabectedin (Yondelis). SMC No. 452/08. July 2011. Second resubmission. Available at: http://www.scottishmedicines.org.uk/files//advice/trabectedin_Yondelis_2ND_RESUBMISSION_FINAL_JUNE_2011_for_website.pdf. Accessed 8 February 2015.

32. Shingler SL, Swinburn P, Lloyd A, Diaz J, Isbell R, Manson S, et al. Elicitation of health state utilities in soft tissue sarcoma. Qual Life Res. 2013 Sep;22(7):1697-706.

33. Soini EJ, García San Andres B, Joensuu T. Economic evaluation of trabectedin in the treatment of metastatic soft-tissue sarcoma (MSTS) in the Finnish setting. Value Health. 2009;12(7):A277.

34. Soini EJ, García San Andres B, Joensuu T. Trabectedin in the treatment of metastatic soft tissue sarcoma: cost-effectiveness, cost-utility and value of information. Ann Oncol. 2011 Jan;22(1):215-23.

35. Villa G, Hernández-Pastor LJ, Guix M, Lavernia J, Cuesta M. Cost-effectiveness analysis of pazopanib in second-line treatment of advanced soft tissue sarcoma in Spain. Clin Transl Oncol. 2015 Jan;17(1):24-33.

Figure S-2. Economic Model Structure and Health-Utility Estimates

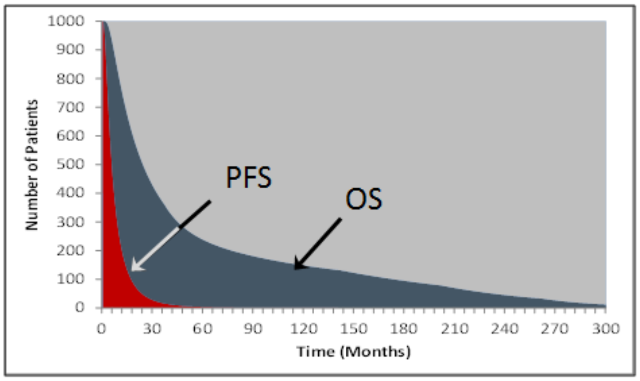

Line of Therapy		Health state	Utility weight, mean (SE)	Source
1 Line		Progression free	0.72 (0.08)	Reichardt et al., 2012
		Progressed	0.56 (0.05)	Reichardt et al., 2012
≥2 Line		Progression free	0.68 (0.02)	Delea et al., 2014
		Progressed	0.42 (0.02)	Delea et al., 2014
Any line		Progression free	0.70[a]	Average of 1L and ≥2L
		Progressed	0.51[a]	Average of 1L and ≥2L
All		Dead	0.00	By definition

OS = overall survival; PFS = progression-free survival; SE = standard error. Note that PFS and OS curves shown are illustrative only.

[a] Calculated as the weighted average of the estimates for first-line and second- or later-line treatment, based on the proportion of the population entering the model and receiving first-line or later-line therapy. The estimate of uncertainty was calculated in the model to account for uncertainty in values for first-line and second- or later-line therapy.

Table S-3. Summary of Base-Case Incidence of Adverse Events Applied in the Economic Model

Adverse Event	Incidence													
	Olara+Dox		Dox[a]		AIM		GemDoc (GeDDiS)		GemDoc (Maki)		PLD		MAID	
	%	n/N[b]	%	n/N[b]	%	n/N[b]	%	n/N[b]	%	n/N[b]	%	n/N[b]	%	n/N[b]
**Grade ≥ 3 events**														
Abdominal pain	3.1	2/64	0.0	0/65	0.4	1/224	0.0[c]	0/126	1.4	1/73	0.0[c]	0/50	0.0[c]	0/80
Anemia	12.5	8/64	9.2	6/65	34.8	78/224	6.3	8/126	6.8	5/73	10.0	5/50	60.0	48/80
Asthenia (fatigue)	9.4	6/64	3.1	2/65	7.6	17/224	13.5	17/126	16.4	12/73	0.0[c]	0/50	8.8	7/80
Back pain	3.1	2/64	0.0	0/65	0.4	1/224	0.0[c]	0/126	1.4	1/73	0.0[c]	0/50	0.0[c]	0/80
Cough	0.0	0/64	0.0	0/65	0.0	0/224	0.0[c]	0/126	1.4	1/73	4.0	2/50	0.0[c]	0/80
Diarrhea	3.1	2/64	0.0	0/65	3.1	7/224	7.9	10/126	1.4	1/73	0.0	0/50	0.0[c]	0/80
Dyspnea	0.0	0/64	1.5	1/65	4.9	11/224	4.0	5/126	0.0[c]	0/73	4.0	2/50	0.0[c]	0/80

Adverse Event	Incidence													
	Olara+Dox		Dox[a]		AIM		GemDoc (GeDDiS)		GemDoc (Maki)		PLD		MAID	
	%	n/N[b]	%	n/N[b]	%	n/N[b]	%	n/N[b]	%	n/N[b]	%	n/N[b]	%	n/N[b]
Febrile neutropenia	12.5	8/64	13.8	9/65	46.0	103/224	11.9	15/126	5.5	4/73	2.0	1/50	56.0	45/80
GI hemorrhage	0.0	0/64	1.5	1/65	0.0[c]	0/224	0.0[c]	0/126	2.7	2/73	0.0[c]	0/50	1.3	1/80
GI perforation	1.6	1/64	0.0	0/65	0.0	0/224	0.0[c]	0/126	1.4	1/73	0.0[c]	0/50	0.0[c]	0/80
Hypokalemia	3.1	2/64	4.6	3/65	0.0	0/224	0.0[c]	0/126	1.4	1/73	0.0[c]	0/50	0.0[c]	0/80
Infection	7.8	5/64	10.8	7/65	17.9	40/224	0.0[c]	0/126	0.0[c]	0/73	4.0	2/50	8.8	7/80
Leukopenia	21.9	14/64	6.2	4/65	43.3	97/224	7.1	9/126	0.0[c]	0/73	2.0	1/50	0.0[c]	0/80
Lymphopenia	3.1	2/64	1.5	1/65	0.0[c]	0/224	0.0[c]	0/126	5.5	4/73	0.0[c]	0/50	0.0[c]	0/80
Mucosal inflammation	3.1	2/64	4.6	3/65	5.8	13/224	1.6	2/126	1.4	1/73	4.0	2/50	6.3	5/80
Nausea/ vomiting	1.6	1/64	3.1	2/65	12.1	27/224	2.4	3/126	5.5	4/73	0.0	0/50	20.0	16/80
Neutropenia	43.8	28/64	16.9	11/65	41.5	93/224	19.0	24/126	16.4	12/73	6.0	3/50	85.0	68/80

Adverse Event	Incidence													
	Olara+Dox		Dox[a]		AIM		GemDoc (GeDDiS)		GemDoc (Maki)		PLD		MAID	
	%	n/N[b]	%	n/N[b]	%	n/N[b]	%	n/N[b]	%	n/N[b]	%	n/N[b]	%	n/N[b]
Pleural effusion	1.6	1/64	1.5	1/65	0.9	2/224	0.0[c]	0/126	1.4	1/73	0.0[c]	0/50	0.0[c]	0/80
Thrombocyto-penia	9.4	6/64	7.7	5/65	33.5	75/224	0.0[c]	0/126	39.7	29/73	0.0	0/50	33.8	27/80
**Grade 1-2 events**														
Diarrhea	31.3	20/64	23.1	15/65	27.2	61/224	31.3[d]	N/A	31.3[d]	N/A	16.0	8/50	31.3[d]	N/A
Fatigue	59.4	38/64	66.2	43/65	77.2	173/224	59.4[d]	N/A	59.4[d]	N/A	59.4[d]	N/A	59.4[d]	N/A
Mucositis	50.0	32/64	30.8	20/65	28.1	63/224	50.0[d]	N/A	50.0[d]	N/A	50.0	25/50	50.0[d]	N/A
Nausea	71.9	46/64	49.2	32/65	78.1	175/224	71.9[d]	N/A	71.9[d]	N/A	64.0	32/50	71.9[d]	N/A
Vomiting	45.3	29/64	18.5	12/65	55.8	125/224	45.3[d]	N/A	45.3[d]	N/A	32.0	16/50	45.3[d]	N/A

Adverse Event	Incidence													
	Olara+Dox		Dox[a]		AIM		GemDoc (GeDDiS)		GemDoc (Maki)		PLD		MAID	
	%	n/N[b]	%	n/N[b]	%	n/N[b]	%	n/N[b]	%	n/N[b]	%	n/N[b]	%	n/N[b]
Sources	JGDG study, Tap et al. [3]; Eli Lilly data on file [4, 5]				Judson et al. [6]		Seddon et al. [7]		Maki et al. [8]		Judson et al. [9]		Fayette et al. [10]	

AE = adverse event; AIM = ifosfamide + doxorubicin + mesna; Dox = doxorubicin; DTIC = dacarbazine; GI = gastrointestinal; GemDoc = gemcitabine + docetaxel; MAID = mesna + doxorubicin + ifosfamide + DTIC; Olara = olaratumab; Olara+Dox = olaratumab + doxorubicin; PLD = pegylated liposomal doxorubicin (Doxil); PSA = probabilistic sensitivity analysis.

[a] Excluding AE occurring following initiation of post-progression Olara monotherapy in the Dox arm.

[b] Measure of uncertainty in the PSA (beta distribution).

[c] AE rate not reported; assumed to be 0%.

[d] Assumed to have the same incidence as in Olara+Dox arm.

Table S-4. Summary of Mean Number of Adverse Events per Patient Having the Event

Adverse Event	Mean Events per Patient[a]			
	Olara+Dox		Dox	
	Mean	SE[b]	Mean	SE[b]
**Grade ≥ 3 events**				
Abdominal pain[c]	1.00	0.20	N/A	N/A
Anemia	1.75	0.25	1.00	0.20
Asthenia (fatigue)	1.17	0.17	1.00	0.20
Back pain	1.00	0.20	N/A	N/A
Cough	N/A	N/A	1.00	0.20
Diarrhea	1.00	0.20	N/A	N/A
Dyspnea	N/A	N/A	1.00	0.20
Febrile neutropenia	1.00	0.20	1.11	0.11
GI hemorrhage[c]	1.00	0.20	N/A	N/A
GI perforation	1.00	0.20	N/A	N/A
Hypokalemia[d]	1.61	0.28	1.09	0.09
Infection	1.20	0.20	1.14	0.14
Leukopenia[d]	1.61	0.28	1.09	0.09
Lymphopenia[d]	1.61	0.28	1.09	0.09
Mucosal inflammation	1.00	0.20	1.00	0.20
Nausea/vomiting	1.00	0.20	1.00	0.20
Neutropenia	1.61	0.28	1.09	0.09

Adverse Event	Mean Events per Patient[a]			
	Olara+Dox		Dox	
	Mean	SE[b]	Mean	SE[b]
Pleural effusion	N/A	N/A	1.00	0.20
Thrombocytopenia	2.29	0.65	1.20	0.20
**Grade 1-2 events**				
Diarrhea	1.00	0.20	1.00	0.20
Fatigue	1.16	0.23	1.02	0.20
Mucositis	1.03	0.21	1.15	0.23
Nausea	1.02	0.20	1.06	0.21
Vomiting	1.00	0.20	1.00	0.20

AIM = ifosfamide + doxorubicin + mesna; Dox = doxorubicin; GI = gastrointestinal; GemDoc = gemcitabine + docetaxel; MAID = mesna + doxorubicin + ifosfamide + DTIC; N/A = not applicable; Olara+Dox = olaratumab + doxorubicin; PLD = pegylated liposomal doxorubicin (Doxil); PSA = probabilistic sensitivity analysis; SE = standard error.

[a] Mean among patients having at least one event of that type. No data were available for AIM, PLD, MAID and GemDoc; the mean was assumed to be equal to that for Olara+Dox.

[b] Measure of uncertainty in the PSA (normal distribution).

[c] Data not available, assumed to be the same as GI (perforation).

[d] Data not available assumed to be the same as neutropenia.

Sources: Tap et al. [3]; Eli Lilly data on file [4, 5].

Table S-5. Summary of Base-Case Grade ≥ 3 Adverse-Event Costs and Utility Decrements Applied in the Economic Model

Grade ≥ 3 Adverse Event	Cost (US $)[a]	Source	Utility Decrement		Duration (Weeks)[b]		Source for the Utility Decrement
	Mean[c]	HCUP ICD-9 Code	Mean	SE[d]	Mean	SE[e]	
Abdominal pain[f]	6,481	789.00	0.118	0.02	0.1	0.0	Assumption (same as GI perforation)
Anemia	6,653	285.90	0.119	0.02	5.7	1.1	Amdahl et al. [11]; referenced to Swinburn et al. [12] (anemia/hemoglobin)
Asthenia (fatigue)	6,959	780.79	0.262	0.03	0.6	0.1	Shingler et al. [13] (fatigue grade 3/4)
Back pain	8,343	729.10	0.236	0.03	0.6	0.1	Shingler et al. [13] (pain grade 3/4)
Cough[g]	5,297	786.20	0.242	0.03	1.0	0.2	Shingler et al. [13] (dyspnea grade 3/4)
Diarrhea	7,531	787.91	0.327	0.03	1.0	0.2	Shingler et al. [13] (diarrhea grade 3/4)
Dyspnea	6,317	786.05	0.242	0.03	1.0	0.2	Shingler et al. [13] (dyspnea grade 3/4)
Febrile neutropenia	19,494	288.04	0.090	0.02	0.8	0.2	Amdahl et al. [11]; referenced to Nafees et al. [14]
GI hemorrhage[f]	10,063	578.90	0.118	0.02	0.1	0.0	Assumption (same as GI perforation)

Grade ≥ 3 Adverse Event	Cost (US $)[a]	Source	Utility Decrement		Duration (Weeks)[b]		Source for the Utility Decrement
	Mean[c]	HCUP ICD-9 Code	Mean	SE[d]	Mean	SE[e]	
GI perforation	29,411	569.83	0.118	0.02	0.1	0.0	Nintedanib NICE appraisal (TA379)) based on Leontiadis et al. (2007)
Hypokalemia[h]	6,393	276.80	0.09	0.02	1.4	0.3	Assumption (same as neutropenia)
Infection	14,544	995.91	0.090	0.02	1.3	0.3	Assumption (same as febrile neutropenia)
Leukopenia[h]	7,411	288.50	0.09	0.03	1.4	0.3	Assumption (same as neutropenia)
Lymphopenia[h]	7,196	288.80	0.09	0.02	1.4	0.3	Assumption (same as neutropenia)
Mucosal inflammation	9,494	528.90	0.151	0.03	1.0	0.2	Lloyd et al. [15] (stomatitis grade 3/4)
Nausea/vomiting	6,731	787.01	0.357	0.02	1.0	0.2	Shingler et al. [13] (nausea and vomiting grade 3/4)
Neutropenia	12,187	288.03	0.090	0.02	1.4	0.3	Amdahl et al. [11]; referenced to Nafees et al. [14] (leukopenia, neutropenia, and thrombocytopenia)
Pleural effusion[g]	12,562	511.90	0.236	0.03	1.0	0.2	Shingler et al. [13] (dyspnea grade 3/4)

Grade ≥ 3 Adverse Event	Cost (US $)[a]	Source	Utility Decrement		Duration (Weeks)[b]		Source for the Utility Decrement
	Mean[c]	HCUP ICD-9 Code	Mean	SE[d]	Mean	SE[e]	
Thrombocytopenia	11,848	287.49	0.090	0.02	0.1	0.0	Assumption from Shingler et al. [13]; same as neutropenia

AIM = ifosfamide + doxorubicin + mesna; Dox = doxorubicin; DTIC = dacarbazine; GemDoc = gemcitabine + docetaxel; GI = gastrointestinal; HCUP = Healthcare Cost and Utilization Project; ICD-9 = International Classification of Diseases, 9th Modification; MAID = mesna + doxorubicin + ifosfamide + DTIC; NICE = National Institute for Health and Care Excellence; Olara+Dox = olaratumab + doxorubicin; PLD = pegylated liposomal doxorubicin (Doxil); SE = standard error; UK = United Kingdom; US = United States.

[a] Sources: Tap et al. [3]; Eli Lilly data on file [5]; Agency for Healthcare Research and Quality [16].

[b] Expert opinion (UK Advisory Board Meeting; 12 April 2016). No data were available for AIM, GemDoc (GeDDiS), GemDoc (Maki), PLD, or MAID; the mean was assumed to be equal to that for Olara+Dox and Dox.

[c] The measure of uncertainty was the SE (normal distribution assumed to be 20% of the mean) for individual HCUP ICD-9 codes.

[d] Beta distribution.

[e] Measure of uncertainty assumed to be 20% of the mean.

[f] Assumed to be the same as GI (perforation).

[g] Assumed to be the same as dyspnea.

[h] Assumed to be the same as neutropenia.

Table S-6. Summary of Base-Case Grade 1-2 Adverse-Event Costs and Utility Decrements Applied in the Economic Model

	Cost[b]	Utility Decrement		Duration (Weeks)[a]				
				Olara+Dox		Dox		Source for the Utility
Grade 1-2 Adverse Event	Mean	Mean	SE	Mean	SE[c]	Mean	SE[c]	Decrement
Diarrhea	N/A	0.060	0.010	1.5	0.150	1.5	0.150	Beusterien et al. [17] (flu-like syndrome)
Fatigue	N/A	0.090	0.010	3.3	0.330	3.3	0.330	Beusterien et al. [17]
Mucositis	N/A	0.100	0.020	3.1	0.310	3.1	0.310	Beusterien et al. [17] (stomatitis)
Nausea	N/A	0.070	0.010	3.0	0.300	3.0	0.300	Beusterien et al. [17]
Vomiting	N/A	0.070	0.010	1.5	0.150	1.5	0.150	Beusterien et al. [17]

Dox = doxorubicin; N/A = not applicable; Olara+Dox = olaratumab + doxorubicin; SE = standard error; UK = United Kingdom.

[a] Source: UK Advisory Board Meeting, April 12, 2016.

[b] The cost of grade 1-2 adverse events was assumed to be negligible and was excluded from the model.

[c] Measure of uncertainty assumed to be 20% of the mean.

Table S-7. Summary of Base-Case Variables Applied in the Economic Model

Variable	Value	Measurement of Uncertainty (Distribution)	Reference
Line of therapy investigated	Any line	N/A	N/A
Discount rate: costs	3.0%	N/A	Gold et al. [18]
Discount rate: outcomes	3.0%	N/A	Gold et al. [18]
Mean age (years)	58	SE = 1.09 (normal)	Eli Lilly data on file [4]
Mean BSA (m^2)	2.0	SE = 0.04 (normal)	Eli Lilly data on file [4]
Mean weight (kg)	85.8	SE = 2.83 (normal)	Eli Lilly data on file [4]
Percentage female	56%	n/N = 74/133 (beta)	Tap et al. [3]
Percentage of first-line patients in any-line analysis	71%	n/N = 371/520 (beta)	SEER Medicare study (Eli Lilly data on file [19])
PFS (investigator assessed): Olara+Dox vs. Dox/PLD/GemDoc (Maki)	Kaplan-Meier	SE (normal)	Tap et al.[3] and Eli Lilly data on file [20]
PFS (investigator assessed): Olara+Dox vs. AIM/GemDoc (GeDDiS)/MAID	HR	SE (normal)	Bertwistle et al. [22]

Variable	Value	Measurement of Uncertainty (Distribution)	Reference
OS: Olara+Dox vs. Dox/GemDoc (Maki)/PLD up to last mortality event in Olara+Dox arm of JGDG trial (32 months)	Gamma, arms together	Variance-covariance matrix (Cholesky decomposition)	Eli Lilly data on file [20]
OS: Olara+Dox vs. AIM/GemDoc (GeDDiS)/MAID	HR	SE (normal)	Bertwistle et al. [22]
OS prediction for Dox beyond last mortality event in Olara+Dox arm of JGDG trial (32 months)	Dox gamma function fitted to the JGDG data	Variance-covariance matrix (Cholesky decomposition)	Eli Lilly data on file [20]
Treatment effect after trial follow-up	None (HR = 1.00)	Fixed	Assumption
Age-specific mortality rate	US general population mortality rates by age and sex	N/A[a]	Centers for Disease Control and Prevention [23]

Variable	Value	Measurement of Uncertainty (Distribution)	Reference
Age-specific mortality, HR for STS vs. general population	5.19	SE = 1.038[b] (normal)	
Response rates (CR/PR)	N/A[c]	N/A	
**Health-state utility values[d]**			
Progression-free, first line	0.720	SE = 0.075 (beta)	Reichardt et al. [24]
Progressed, first line	0.560	SE = 0.051 (beta)	Reichardt et al. [24]
Progression-free, ≥ second line	0.678	SE = 0.024 (beta)	Delea et al. [25]
Progressed, ≥ second line	0.425[e]	SE = 0.024 (beta)[e]	Delea et al. [25]
**Mean dose (any line of therapy analysis): Olara+Dox**			
Olara (mg/kg)	14.0	SE = 0.074 (normal)	Eli Lilly data on file [5]
Dox (mg/m^2)	73.7	SE = 0.341 (normal)	Eli Lilly data on file [5]
Dex (mg/m^2) (mean among patients receiving Dex)	707.0	SE = 7.034 (normal)	Eli Lilly data on file [5]

Variable	Value	Measurement of Uncertainty (Distribution)	Reference
Percentage receiving Dex	59%	N/A	Eli Lilly data on file [5]
**Mean dose (any line of therapy analysis): Dox**			
Dox (mg/m^2)	74.7	SE = 0.296 (normal)	Eli Lilly data on file [5]
Dex (mg/m^2) (mean among patients receiving Dex)	725.8	SE = 6.520 (normal)	Eli Lilly data on file [5]
Percentage receiving Dex	45%	N/A	Eli Lilly data on file [5]
**Mean dose (any line of therapy analysis): AIM**			
Ifo (mg/m^2)	2500	SE = 500[b] (normal)	Judson et al. [6]
Dox (mg/m^2)	25	SE = 5[b] (normal)	Judson et al. [6]
Mesna (mg/m^2)	2000	SE = 400[b] (normal)	Assumption
Pegfilgrastim (mg)	6	SE = 1.20[b] (normal)	Judson et al. [6]
Dex (mg/m^2)	250	SE = 50[b] (normal)	FDA [26]

Variable	Value	Measurement of Uncertainty (Distribution)	Reference
**Mean dose (any line of therapy analysis): GemDoc (GeDDiS)**			
Gem (mg/m^2)	675.0	SE = 135.0[b] (normal)	Seddon et al. [7]
Doc (mg/m^2)	75.0	SE = 15.0[b] (normal)	Seddon et al. [7]
**Mean dose (any line of therapy analysis): GemDoc (Maki)**			
Gem (mg/m^2)	900	SE = 180.0[b] (normal)	Maki et al. [8]
Doc (mg/m^2)	100	SE = 20.0[b] (normal)	Maki et al. [8]
Pegfilgrastim (mg)	6	SE = 1.2[b] (normal)	Maki et al. [8]
**Mean dose (any line of therapy analysis): PLD**			
PLD (mg/m^2)	50	SE = 10[b] (normal)	Judson et al. [9]
**Mean dose (any line of therapy analysis): MAID**			
Mesna (mg/m^2)	2500.0	SE = 500.0[b] (normal)	Bui-Nguyen et al. [27]
Dox (mg/m^2)	20.0	SE = 4.0[b] (normal)	Bui-Nguyen et al. [27]
Ifo (mg/m^2)	2500.0	SE = 500.0[b] (normal)	Bui-Nguyen et al. [27]
DTIC (mg/m^2)	300.0	SE = 60.0[b] (normal)	Bui-Nguyen et al. [27]

Variable	Value	Measurement of Uncertainty (Distribution)	Reference
Dex (mg/m^2)	200	SE = 40.0[b] (normal)	Assumption: 10 times the Dox dose
**Mean number of administrations (any line of therapy analysis): Olara+Dox**			
Olara	19.4	SE = 2.184 (normal)	Eli Lilly data on file [5]
Dox	5.7	SE = 0.320 (normal)	Eli Lilly data on file [5]
Dex (mean among patients receiving Dex)	3.6	SE = 0.144 (normal)	Eli Lilly data on file [5]
**Mean number of administrations (any line of therapy analysis): Dox**			
Dox	4.40	SE = 0.331 (normal)	Eli Lilly data on file [5]
Dex (mean among patients receiving Dex)	3.14	SE = 0.209 (normal)	Eli Lilly data on file [5]

Variable	Value	Measurement of Uncertainty (Distribution)	Reference
**Mean number of administrations (any line of therapy analysis): AIM**			
Ifo	17.7	SE = 3.535[b] (normal)	Judson et al. [6, 28][f]
Dox	13.3	SE = 2.651[b] (normal)	Judson et al. [6, 28][f]
Mesna	17.7	SE = 3.535[b] (normal)	Judson et al. [6, 28][f]
Pegfilgrastim	4.4	SE = 0.884[b] (normal)	Judson et al. [6, 28][f]
Dex	5.7	SE = 1.138[b] (normal)	Assumption (3 times the mean number of Dex administrations in the Dox arm in the JGDG study for patients receiving cycles 5 and 6)

Variable	Value	Measurement of Uncertainty (Distribution)	Reference
**Mean number of administrations (any line of therapy analysis): GemDoc (GeDDiS)**			
Gem	8.2	SE = 1.64[b] (normal)	Assumed number of cycles = Dox (up to 6 cycles), estimated from Judson et al. [28][g]
Doc	4.1	SE = 0.82[b] (normal)	Assumed number of cycles = Dox (up to 6 cycles), estimated from Judson et al. [28][g]
**Mean number of administrations (any line of therapy analysis): GemDoc (Maki)**			
Gem	8.0	SE = 1.60[b] (normal)	Maki et al. [8][h]
Doc	4.0	SE = 0.80[b] (normal)	Maki et al. [8][h]
Pegfilgrastim	4.0	SE = 0.80[b] (normal)	Maki et al. [8][h]

Variable	Value	Measurement of Uncertainty (Distribution)	Reference
**Mean number of administrations (any line of therapy analysis): PLD**			
PLD	3.4	SE = 0.67[b] (normal)	Judson et al. [9]
**Mean number of administrations (any line of therapy analysis): MAID**			
Mesna	13.3	SE = 2.651[b] (normal)	Judson et al. [6]
Dox	13.3	SE = 2.651[b] (normal)	Judson et al. [6]
Ifo	13.3	SE = 2.651[b] (normal)	Judson et al. [6]
DTIC	13.3	SE = 2.651[b] (normal)	Judson et al. [6]
Dex	5.7	SE = 1.138[b] (normal)	FDA [26]
**Drug-administration costs**			
Olara + Dox, day 1	379.76	SE = see footnote[i] (normal)	Essential RBRVS [29] (HCPCS 96409, 96413)
Olara + Dox + Dex, day 1	476.21	SE = see footnote[i] (normal)	Essential RBRVS [29] (HCPCS 96409, 96413, 96417, 96415)

Variable	Value	Measurement of Uncertainty (Distribution)	Reference
Olara, day 1	208.79	SE = see footnote[i] (normal)	Essential RBRVS [29] (HCPCS 96413)
Olara, day 8	208.79	SE = see footnote[i] (normal)	Essential RBRVS [29] (HCPCS 96413)
Dox, day 1	170.98	SE = see footnote[i] (normal)	Essential RBRVS [29] (HCPCS 96409)
Dox + Dex, day 1	379.76	SE = see footnote[i] (normal)	Essential RBRVS [29] (HCPCS 96409, 96413)
AIM (A+I+M), day 1	572.11	SE = see footnote[i] (normal)	Essential RBRVS [29] (HCPCS 96409, 96411, 96413, 96417)
AIM (A+I+M), day 2	572.11	SE = see footnote[i] (normal)	Essential RBRVS [29] (HCPCS 96409, 96411, 96413, 96417)
AIM (A+I+M), day 3	572.11	SE = see footnote[i] (normal)	Essential RBRVS [29] (HCPCS 96409, 96411, 96413, 96417)
AIM (I+M), day 4	476.21	SE = see footnote[i] (normal)	Essential RBRVS [29] (HCPCS 96409, 96413, 96417)
Pegfilgrastim, day 5	115.08	SE = see footnote[i] (normal)	Essential RBRVS [29] (HCPCS 96401)

Variable	Value	Measurement of Uncertainty (Distribution)	Reference
AIM + Dex, day 1	668.56	SE = see footnote[i] (normal)	Essential RBRVS [29] (HCPCS 96409, 96411, 96413, 96417, 96415)
AIM + Dex, day 2	668.56	SE = see footnote[i] (normal)	Essential RBRVS [29] (HCPCS 96409, 96411, 96413, 96417, 96415)
AIM + Dex, day 3	668.56	SE = see footnote[i] (normal)	Essential RBRVS [29] (HCPCS 96409, 96411, 96413, 96417, 96415)
Gem, day 1	252.63	SE = see footnote[i] (normal)	Essential RBRVS [29] (HCPCS 96413, 96415)
Gem + Doc, day 8	349.08	SE = see footnote[i] (normal)	Essential RBRVS [29] (HCPCS 96413, 96417, 96415)
Pegfilgrastim, day 9	115.08	SE = see footnote[i] (normal)	Essential RBRVS [29] (HCPCS 96401)
PLD, day 1	208.79	SE = see footnote[i] (normal)	Essential RBRVS [29] (HCPCS 96413)
MAID, day 1	772.13	SE = see footnote[i] (normal)	Essential RBRVS [29] (HCPCS 96409, 96413, 96417, 96415, 96360, 96361)

Variable	Value	Measurement of Uncertainty (Distribution)	Reference
MAID, day 2	772.13	SE = see footnote[i] (normal)	Essential RBRVS [29] (HCPCS 96409, 96413, 96417, 96415, 96360, 96361)
MAID, day 3	772.13	SE = see footnote[i] (normal)	Essential RBRVS [29] (HCPCS 96409, 96413, 96417, 96415, 96360, 96361)
MAID + Dex, day 1	868.58	SE = see footnote[i] (normal)	Essential RBRVS [29] (HCPCS 96409, 96413, 96417, 96415, 96360, 96361)
MAID + Dex, day 2	868.58	SE = see footnote[i] (normal)	Essential RBRVS [29] (HCPCS 96409, 96413, 96417, 96415, 96360, 96361)
MAID + Dex, day 3	868.58	SE = see footnote[i] (normal)	Essential RBRVS [29] (HCPCS 96409, 96413, 96417, 96415, 96360, 96361)
**Cardiac monitoring**			

Variable	Value	Measurement of Uncertainty (Distribution)	Reference
Percentage of Dox patients receiving cardiac monitoring	100%	SE = 10%[b] (normal, truncated at 1)	Assumption

Variable	Value	Measurement of Uncertainty (Distribution)	Reference
**Cost of cardiac-monitoring tests (US $)**			
MUGA	366.06	SE = 73.21[b] (normal)	Essential RBRVS [29] (HCPCS 78472)
Echocardiography	352.36	SE = 70.47[b] (normal)	Essential RBRVS [29] (HCPCS 93306)
**Cardiac-monitoring tests**			
Echocardiography	One every second Dox cycle	SE = 0.160 (normal)	Clinical opinion (personal communication; UK clinical expert; October 21, 2015, follow-up questions)
MUGA	One per Dox cycle	SE = 0.320 (normal)	Clinical opinion (personal communication; UK clinical expert; October 21, 2015, follow-up questions)

Variable	Value	Measurement of Uncertainty (Distribution)	Reference
**Regular follow-up visits and imaging costs (US $)**			
Outpatient visit and physical examination	223.04	SE = 44.61[b] (normal)	Essential RBRVS [29] (HCPCS 99215)
Computerized tomography scan	352.78	SE = see footnote[j]	Essential RBRVS [29] (HCPCS average computerized tomography with dye)
Positron emission tomography	1,072.98	SE = 214.60[b] (normal)	Essential RBRVS [29] (HCPCS 78813)
MRI	584.29	SE = see footnote[k]	Essential RBRVS [29] (HCPCS average MRI without dye)
**Resources for each regular follow-up visit**			
Outpatient visit and physical examination	100%	Fixed	Assumption
Computerized tomography scan	92%	n/N = 183/199 (beta)	UK observational study, Eli Lilly data on file [30]
Positron emission tomography	9%	n/N = 18/199 (beta)	UK observational study, Eli Lilly data on file [30]

Variable	Value	Measurement of Uncertainty (Distribution)	Reference
MRI	14%	n/N = 27/199 (beta)	UK observational study, Eli Lilly data on file [30]
**Frequency of follow-up visits (number of months between each visit)**			
0-5 years	3	SE = 0.6[b] (normal)	Assumption based on clinical opinion (personal communication; UK clinical expert; October 21, 2015)
5-7 years	6	SE = 1.2[b] (normal)	
After 7 years	12	SE = 2.4[b] (normal)	
**Total cost of active therapy after first progression (any line of therapy analysis)**			
**Olara+Dox**			
Total drug cost (US $)	5,418	See footnote[l]	Eli Lilly data on file [5]
Total administration cost (US $)	3,972	See footnote[m]	Eli Lilly data on file [5]

Variable	Value	Measurement of Uncertainty (Distribution)	Reference
Total AE costs (US $)	21,304	SE = 4260.8[b] (normal)	Assumption: average of the AE costs estimated by the model
**Dox, AIM, GemDoc (GeDDiS), GemDoc (Maki), PLD, MAID**			
Total drug cost (US $)	5,515	See footnote[l]	Eli Lilly data on file [5]
Total administration cost (US $)	4,044	See footnote[m]	Eli Lilly data on file [5]
Total AE costs (US $)	21,304	SE = 4260.8[b] (normal)	Assumption: average of the AE costs estimated by the model

AE = adverse event; AIM = ifosfamide + doxorubicin + mesna; BSA = body surface area; CR = complete response; Dex = dexrazoxane; Doc = docetaxel; Dox = doxorubicin; DTIC = dacarbazine; FDA = Food and Drug Administration; Gem = gemcitabine; GemDoc = gemcitabine + doxorubicin; HCPCS = Healthcare Common Procedure Coding System; HR = hazard ratio; Ifo = ifosfamide; MAID = mesna + doxorubicin + ifosfamide + dacarbazine; MRI = magnetic resonance imagining; MUGA = multigated acquisition scan; N/A = not applicable; NHSRC = National Health Service Reference Costs, 2014-2015; NMA = network meta-analysis; Olara = olaratumab; Olara+Dox = olaratumab + doxorubicin; OS = overall survival; PFS = progression-free survival; PLD = pegylated liposomal doxorubicin (Doxil); PR = partial response; PSA = probabilistic sensitivity analysis; RBRVS = resource-based relative value scale; SE = standard error; SEER = Surveillance, Epidemiology, and End Results; STS = soft tissue sarcoma; UK = United Kingdom; US = United States.

[a] Mortality rates by age and sex are not sampled in the PSA because the rates were for the general population.

[b] Assumed to be 20% of the mean value.

[c] Used in sensitivity analysis only.

[d] Estimate for any-line analysis was calculated as a weighted average of the estimates for first line and ≥ second line with the percentage of patients receiving first-line treatment.

[e] Calculated from estimate for progression-free and decrement for progressed versus progression-free (0.253). SE (0.024) is for this decrement.

[f] Estimated from planned number of administrations and mean number of cycles estimated from Judson et al. [28].

[g] Assumption based on no significant difference in PFS between GemDoc and Dox (Seddon et al. [7]). Number of cycles was estimated from detailed exposure to treatment data for the AIM arm of the study by Judson et al. [28].

[h] Estimated from planned number of administrations and median number of cycles reported by Maki et al. [8].

[i] The uncertainty (SE) is calculated separately for each HCPCS code and is assumed to be 20% of the mean value.

[j] The uncertainty (SE) is calculated by HCPCS code for each computerized tomography with dye used for the weighted average.

[k] The uncertainty (SE) is calculated by HCPCS code for each MRI without dye used for the weighted average.

[l] The uncertainty around the BSA is included in the calculation of drug costs of each subsequent active systemic treatment.

[m] The uncertainty is included in the drug-administration cost of each subsequent active systemic treatment based on NHSRC.

Figure S-2. Tornado Diagram for Olara+Dox Versus AIM: Change in ICER (US $ per Life Year Saved)

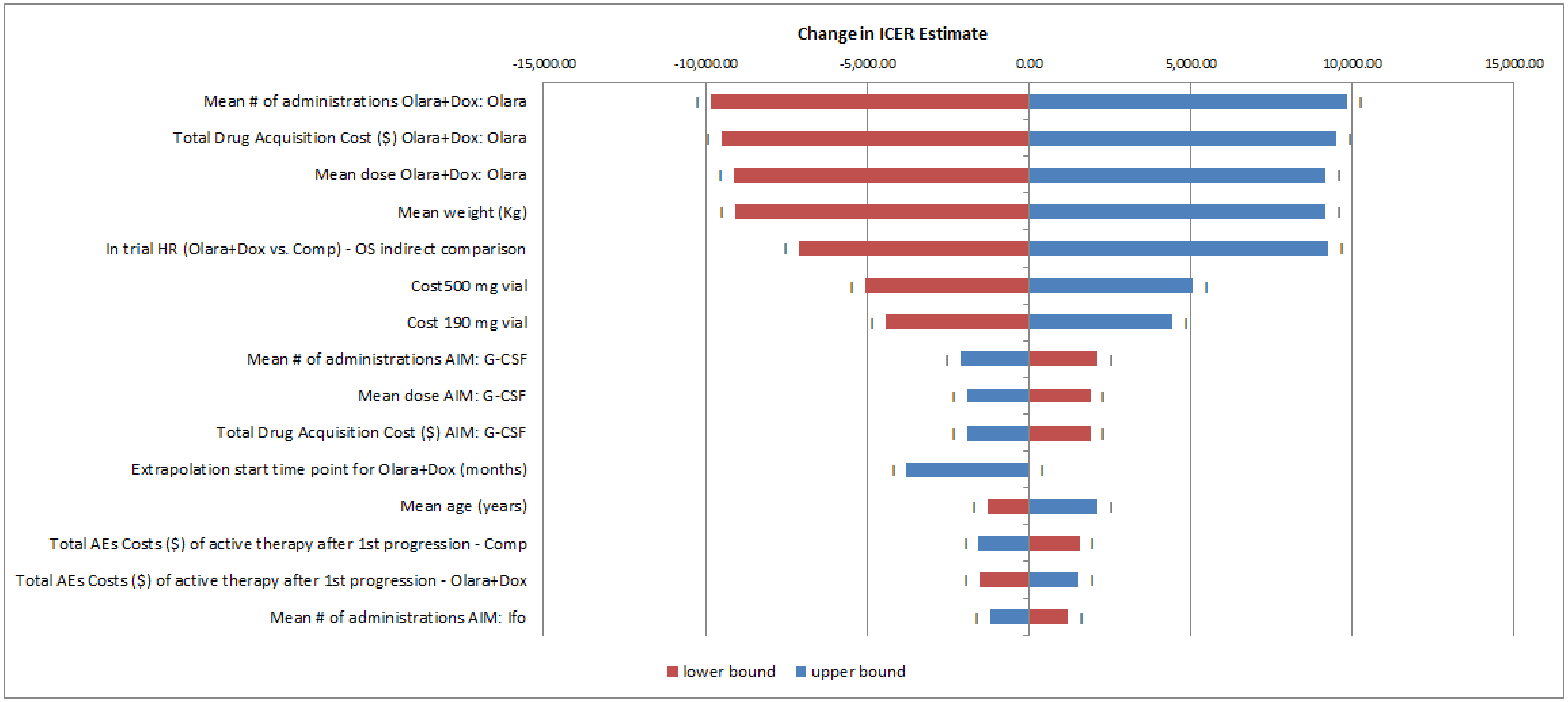

AE = adverse event; AIM = ifosfamide + doxorubicin + mesna; Comp = comparator; G-CSF = granulocyte-colony stimulating factor; HR = hazard ratio; ICER = incremental cost-effectiveness ratio; Ifo = ifosfamide; Olara = olaratumab; Olara+Dox = olaratumab + doxorubicin; OS = overall survival; US = United States.

Note: The quadrant where the ICER falls is shown in the graph at the ends of each bar: I = quadrant 1 (Olara+Dox is more expensive and more effective than the comparator); II = quadrant 2 (Olara+Dox is dominated by the comparator); III = quadrant 3 (Olara+Dox is less expensive and less effective than the comparator); and IV = quadrant 4 (Olara+Dox is dominant over the comparator).

Figure S-3. Tornado Diagram for Olara+Dox Versus GemDoc (GeDDiS): Change in ICER (US $ per LY Saved)

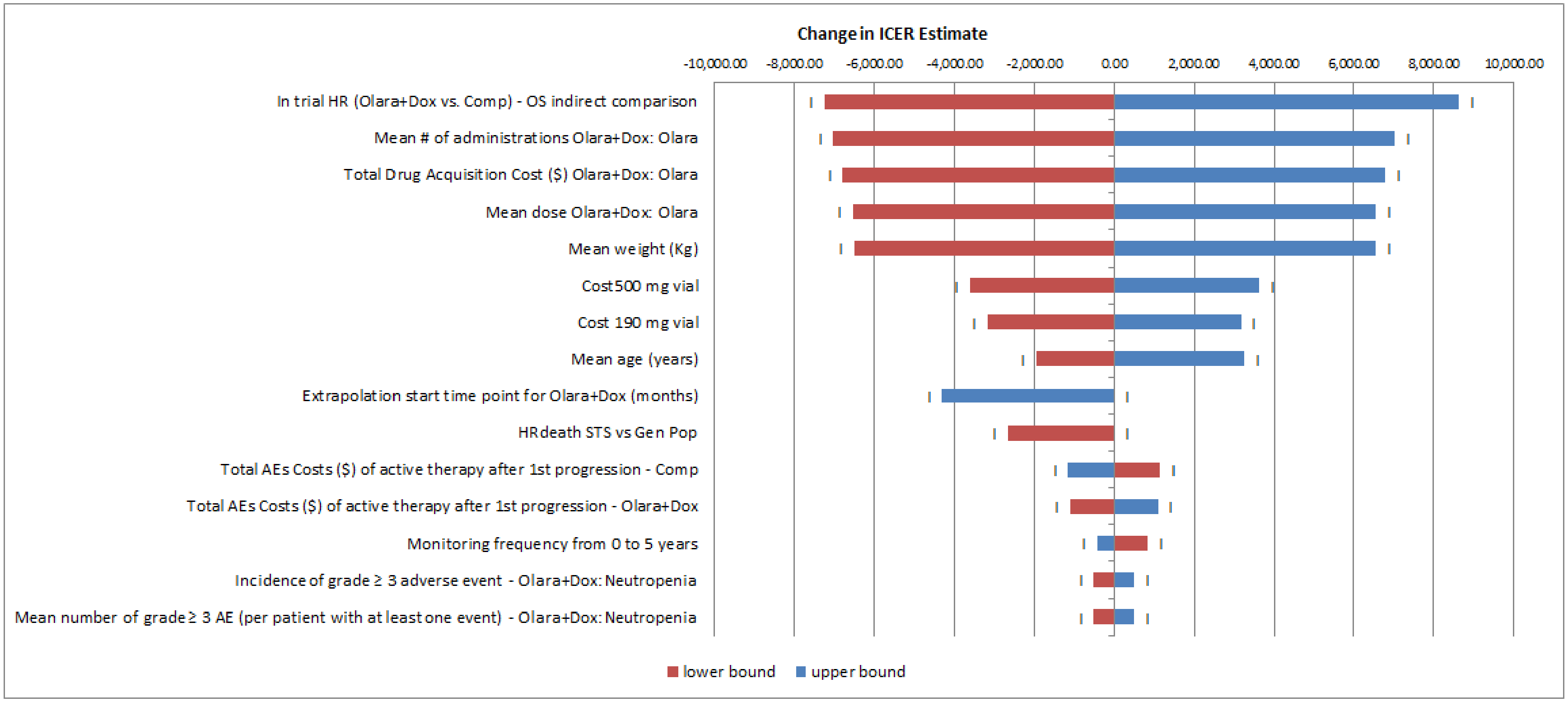

AE = adverse event; Comp = comparator; GemDoc = gemcitabine + docetaxel; Gen Pop = general population; HR = hazard ratio; ICER = incremental cost-effectiveness ratio; LYS = life-year saved; Olara = olaratumab; Olara+Dox = olaratumab + doxorubicin; OS = overall survival; STS = soft tissue sarcoma; US = United States.

Note: The quadrant where the ICER falls is shown in the graph at the ends of each bar: I = quadrant 1 (Olara+Dox is more expensive and more effective than the comparator); II = quadrant 2 (Olara+Dox is dominated by the comparator); III = quadrant 3 (Olara+Dox is less expensive and less effective than the comparator); and IV = quadrant 4 (Olara+Dox is dominant over the comparator).

Figure S-4. Tornado Diagram for Olara+Dox Versus GemDoc (Maki): Change in ICER (US $ per LY Saved)

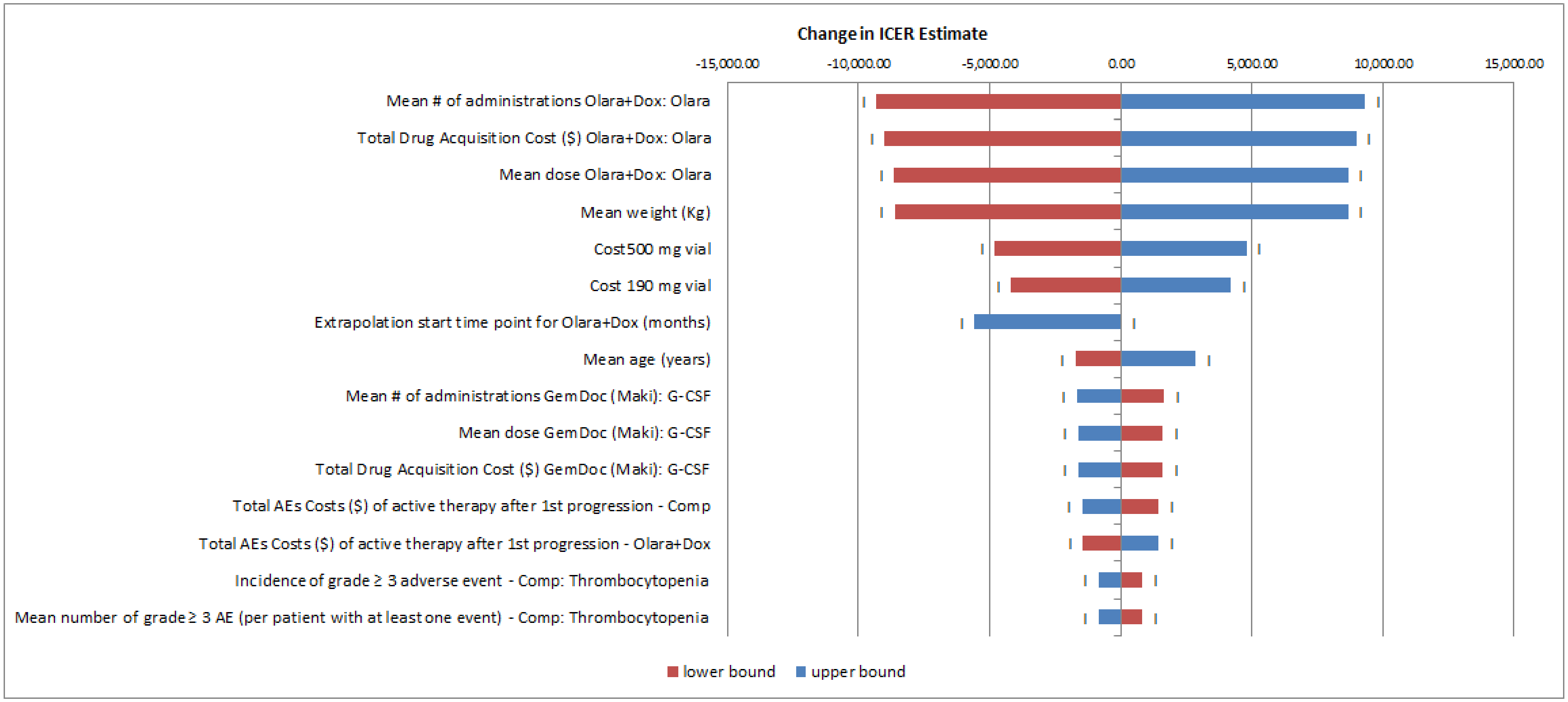

AE = adverse event; Comp = comparator; G-CSF = granulocyte-colony stimulating factor; GemDoc = gemcitabine + docetaxel; ICER = incremental cost-effectiveness ratio; LYS = life-year saved; Olara = olaratumab; Olara+Dox = olaratumab + doxorubicin; US = United States.

Note: The quadrant where the ICER falls is shown in the graph at the ends of each bar: I = quadrant 1 (Olara+Dox is more expensive and more effective than the comparator); II = quadrant 2 (Olara+Dox is dominated by the comparator); III = quadrant 3 (Olara+Dox is less expensive and less effective than the comparator); and IV = quadrant 4 (Olara+Dox is dominant over the comparator).

Figure S-5. Tornado Diagram for Olara+Dox Versus PLD: Change in ICER (US $ per LY Saved)

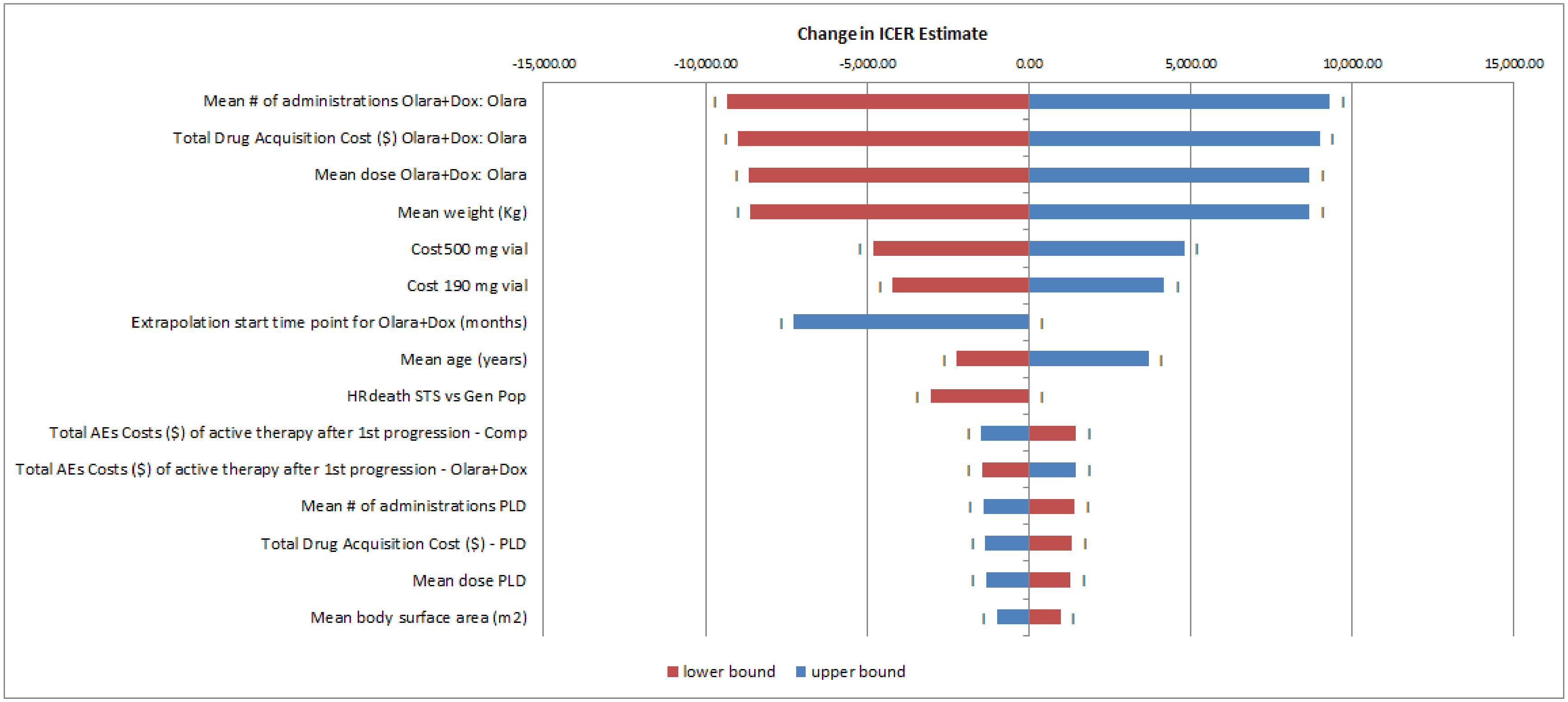

Comp = comparator; Gen Pop = general population; HR = hazard ratio; ICER = incremental cost-effectiveness ratio; LYS = life-year saved; Olara = olaratumab; Olara+Dox = olaratumab + doxorubicin; PLD = pegylated liposomal doxorubicin (Doxil); STS = soft tissue sarcoma; US = United States.

Note: The quadrant where the ICER falls is shown in the graph at the ends of each bar: I = quadrant 1 (Olara+Dox is more expensive and more effective than the comparator); II = quadrant 2 (Olara+Dox is dominated by the comparator); III = quadrant 3 (Olara+Dox is less expensive and less effective than the comparator); and IV = quadrant 4 (Olara+Dox is dominant over the comparator).

Figure S-6. Tornado Diagram for Olara+Dox Versus MAID: Change in ICER (US $ per LY Saved)

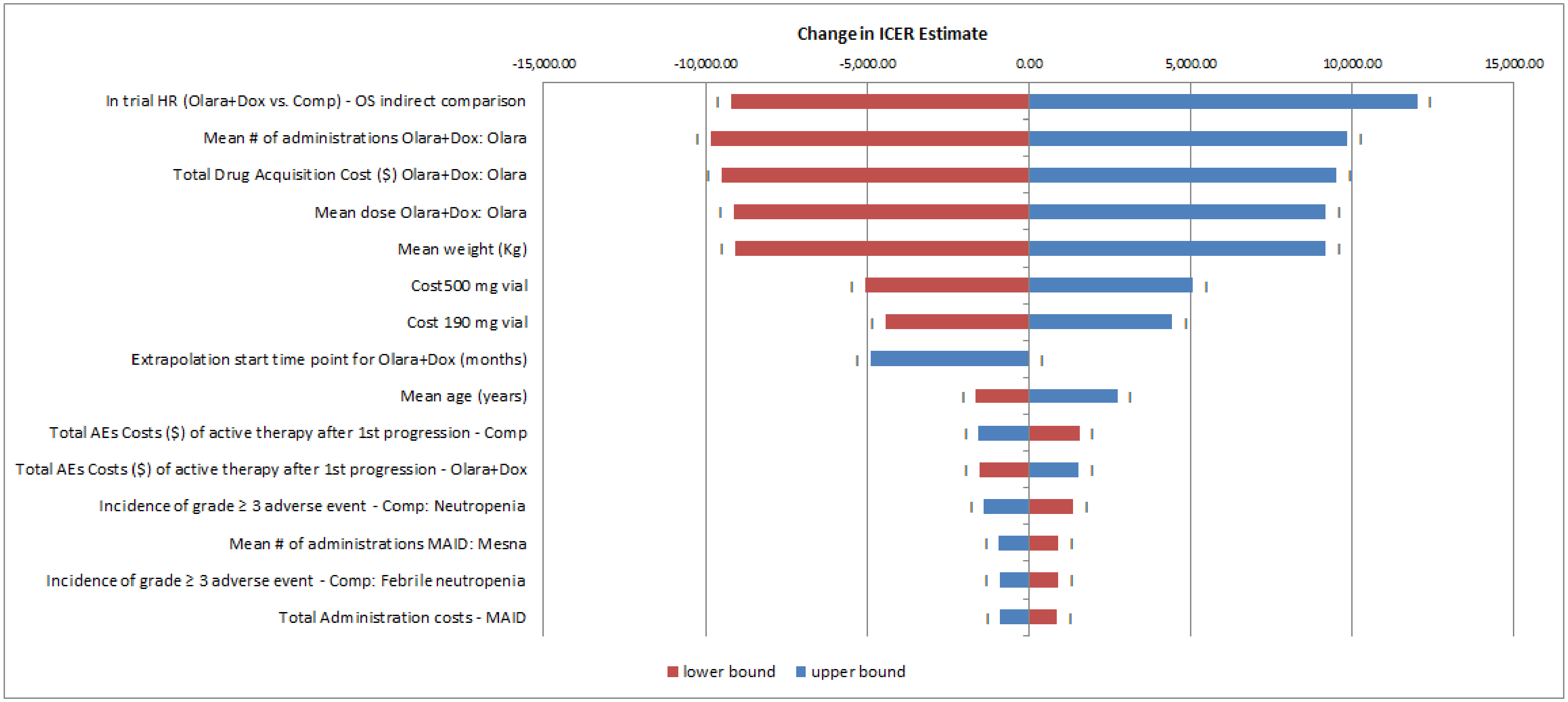

AE = adverse event; Comp = comparator; DTIC = dacarbazine; HR = hazard ratio; LYS = life-year saved; MAID = mesna + doxorubicin + ifosfamide + dacarbazine (DTIC); Olara = olaratumab; Olara+Dox = olaratumab + doxorubicin; OS = overall survival; US = United States.

Note: The quadrant where the ICER falls is shown in the graph at the ends of each bar: I = quadrant 1 (Olara+Dox is more expensive and more effective than the comparator); II = quadrant 2 (Olara+Dox is dominated by the comparator); III = quadrant 3 (Olara+Dox is less expensive and less effective than the comparator); and IV = quadrant 4 (Olara+Dox is dominant over the comparator).

Table S-8. Scenario Analysis Results: Direct Comparison of Olara+Dox With Dox

Parameter	Base Case	Scenario Analysis	Δ Cost (US $)	Δ LY	ICER ($ per LYS)
**Base-case results (all parameters at base-case settings)**			133,653	1.27	105,408
Olara vials	190 mg and 500 mg	500 mg	152,744	1.27	120,464
Discount rates	3.0% for costs and outcomes	0% for costs, 5% for outcomes	134,253	1.16	115,401
		5% for costs, 0% for outcomes	133,338	1.47	90,546
PFS endpoint	Investigator assessed	Blinded, independent, radiological review assessed	133,738	1.27	105,474
PFS function	Kaplan-Meier data	Log-normal (individual treatment arms)	133,621	1.27	105,382
		Weibull (as above)	133,619	1.27	105,381
		Gamma (as above)	133,629	1.27	105,388
		Gompertz (as above)	133,637	1.27	105,395

Parameter	Base Case	Scenario Analysis	Δ Cost (US $)	Δ LY	ICER ($ per LYS)
OS function	Gamma (proportional hazards)	Log-normal (both arms together)	133,510	1.10	121,725
		Weibull (as above)	133,057	0.78	171,593
		Gompertz (as above)	133,127	0.80	165,839
OS extrapolation starting point	32 months	47 months (end of Kaplan-Meier curve)	133,712	1.37	97,883
Treatment effect after trial follow-up	None (HR vs. Dox = 1.00 after 32 months)	Continues indefinitely (HR observed in JGDG study continued to 25 years)	133,620	1.48	90,053
		Tapers to none over 4 years (HR at 32 months = in trial HR, i.e., 0.463)	133,826	1.70	78,669
		Tapers to none over 4 years (HR at 32 months = as end of survival function, i.e., 0.803)	133,699	1.41	94,581

Parameter	Base Case	Scenario Analysis	Δ Cost (US $)	Δ LY	ICER ($ per LYS)
Age-specific mortality: increased risk for STS patients	HR for mSTS vs. general population = 5.19	No increased risk vs. general population (HR = 1.00)	133,661	1.31	102,247
Drug doses<br><br>Olara+Dox arm: Olara<br>Dox<br>Dex<br>Dox arm: Dox<br>Dex	Mean dose administered in JGDG<br>14 mg/kg<br>74 mg/m^2<br>707 mg/m^2<br>75 mg/m^2<br>726 mg/m^2	Planned dose (ignores dose reductions)<br>15 mg/kg<br>75 mg/m^2<br>750 mg/m^2<br>75 mg/m^2<br>750 mg/m^2	141,277	1.27	111,421

Parameter	Base Case	Scenario Analysis	Δ Cost (US $)	Δ LY	ICER ($ per LYS)
Mean total cost of active systemic therapy postprogression (per patient with progression)	JGDG study (observed)	JGDG study (adjusted for follow-up period)	136,936	1.27	107,996

Dex = dexrazoxane; Dox = doxorubicin; HR = hazard ratio; ICER = incremental cost-effectiveness ratio; LY = life-year; LYS = life-year saved; mSTS = metastatic soft tissue sarcoma; Olara = olaratumab; Olara+Dox = olaratumab + doxorubicin; OS = overall survival; PFS = progression-free survival; STS = soft tissue sarcoma; US = United States.

Notes: Δ = delta (difference, Olara+Dox – Dox).

Table S-9 Scenario Analysis Results: Indirect Comparison of Olara+Dox With AIM, GemDoc (GeDDiS), GemDoc (Maki), PLD, and MAID

Parameter	Base Case	Scenario Analysis	Comparator	Δ Cost (US $)	Δ LY	ICER ($ per LYS)
Base-case results (all parameters at base-case settings)			AIM	60,818	1.20	50,701
			GemDoc (GeDDiS)	132,007	1.68	78,679
			GemDoc (Maki)	99,510	1.27	78,480
			PLD	129,059	1.27	101,784
			MAID	78,197	1.20	65,189
G-CSF vial price	$3,898.41	$0	AIM	83,598	1.20	69,692
			GemDoc (GeDDiS)	132,007	1.68	78,679
			GemDoc (Maki)	120,133	1.27	94,745
			PLD	129,059	1.27	101,784
			MAID	78,197	1.20	65,189
Drug doses	Planned doses for all interventions	Dose reduction (%) equals that for Dox arm in	AIM	60,926	1.20	50,791
			GemDoc (GeDDiS)	132,030	1.68	78,692
			GemDoc (Maki)	99,613	1.27	78,561

Parameter	Base Case	Scenario<br><br>Analysis	Comparator	Δ Cost (US $)	Δ LY	ICER<br><br>($ per LYS)
		JGDG (0.35%)	PLD	129,118	1.27	101,831
			MAID	78,231	1.20	65,217

REFERENCES

1. National Institute for Health and Care Excellence (NICE). Single technology appraisal (STA): user guide for company evidence submission template. January 2015. Available at: https://www.nice.org.uk/process/pmg24/resources/single-technology-appraisal-user-guide-for-company-evidence-submission-template-pdf-72286715419333. Accessed November 30, 2016.

2. Drummond MF, Jefferson TO. Guidelines for authors and peer reviewers of economic submissions to the BMJ. BMJ. 2016;313:275-83.

3. Tap WD, Jones RL, Van Tine BA, Chmielowski B, Elias AD, Adkins D, et al. Olaratumab and doxorubicin versus doxorubicin alone for treatment of soft-tissue sarcoma: an open-label phase 1b and randomised phase 2 trial. Lancet. 2016;388:488-97..

4. Eli Lilly and Company (Eli Lilly). Data on file. JGDG clinical study report. I5B-IE-JGDG clinical study report. 21 December 2015.

5. Eli Lilly and Company (Eli Lilly). Data on file. Economic value tool. Additional analyses of the JGDG study data for the economic model. 2016.

6. Judson I, Verweij J, Gelderblom H, Hartmann JT, Schöffski P, Blay J-Y, et al.; European Organisation and Treatment of Cancer–Soft Tissue and Bone Sarcoma Group. Doxorubicin alone versus intensified doxorubicin plus ifosfamide for first-line treatment of advanced or metastatic soft-tissue sarcoma: a randomised controlled phase 3 trial. Lancet Oncol. 2014;15:415-23.

7. Seddon BM, Whelan J, Strauss SJ, Leahy MG, Woll PJ, Cowie F, et al. GeDDiS: a prospective randomized controlled phase III trial of gemcitabine and docetaxel compared with doxorubicin as first-line treatment in previously untreated advanced unresectable or metastatic soft tissue sarcomas (EudraCT 2009-014907-29). J Clin Oncol. 2015;33(suppl). Abstract No. 10500.

8. Maki RG, Wathen JK, Patel SR, Priebat DA, Okuno SH, Samuels B, et al. Randomized phase II study of gemcitabine and docetaxel compared with gemcitabine alone in patients with metastatic soft tissue sarcomas: results of sarcoma alliance for research through collaboration study. J Clin Oncol. 2007;25(19):2755-63.

9. Judson I, Radford JA, Harris M, Blay JY, van Hoesel Q, Le Cesne A, et al. Randomised phase II trial of pegylated liposomal doxorubicin (DOXIL/CAELYX) versus doxorubicin in the treatment of advanced or metastatic soft tissue sarcoma: a study by the EORTC Soft Tissue and Bone Sarcoma Group. Eur J Cancer. 2001 May;37(7):870-7.

10. Fayette J, Penel N, Chevreau C, Blay JY, Cupissol D, Thyss A, et al. Phase III trial of standard versus dose-intensified doxorubicin, ifosfamide and dacarbazine (MAID) in the first-line treatment of metastatic and locally advanced soft tissue sarcoma. Invest New Drugs. 2009;27:482-9.

11. Amdahl J, Manson SC, Isbell R, Chit A, Diaz J, Lewis L, et al. Cost-effectiveness of pazopanib in advanced soft tissue sarcoma in the United Kingdom. Sarcoma. 2014;2014:481071.

12. Swinburn P, Lloyd A, Nathan P, Choueiri TK, Cella D, Neary MP. Elicitation of health state utilities in metastatic renal cell carcinoma. Curr Med Res Opin. 2010 May;26(5):1091-6.

13. Shingler SL, Swinburn P, Lloyd A, Diaz J, Isbell R, Manson S, et al. Elicitation of health-state utilities in soft tissue sarcoma. Qual Life Res. 2013 Sep;22(7):1697-706.

14. Nafees B, Stafford M, Gavriel S, Bhalla S, Watkins J. Health-state utilities for non-small cell lung cancer. Health Qual Life Outcomes. 2008 Oct 21;6:84.

15. Lloyd A, Nafees B, Narewska DS, Watkins J. Health-state utilities for metastatic breast cancer. Br J Cancer. 2006;95:683-90.

16. Agency for Healthcare Research and Quality. Healthcare Cost and Utilization Project (HCUP). 2013. Available at: http://hcupnet.ahrq.gov/. Accessed March 22, 2016.

17. Beusterien KM, Szabo SM, Kotapati S, Mukherjee J, Hoos A, Hersey P, et al. Societal preference values for advanced melanoma health states in the United Kingdom and Australia. Br J Cancer. 2009 Aug 4;101(3):387-9.

18. Gold MR, Siegel JE, Russell LB, Weinstein MC. Cost-effectiveness in health and medicine. New York: Oxford University Press; 1996.

19. Eli Lilly and Company (Eli Lilly). Data on file. United States database study in elderly patients. Project 2015-211 (RTI-HS Project 0303899). 2016.

20. Eli Lilly and Company (Eli Lilly). Data on file. Economic value tool. Survival analyses. Project 2015-208 (RTI-HS Project 0303899). 2016.

21. Eli Lilly and Company (Eli Lilly). Data on file. Comparator clinical trials tool for olaratumab: systematic review and meta-analysis of clinical trials evaluating treatments for advanced soft tissue sarcoma. Project 2014-724a (RTI-HS Project No. 0303899).2016.

22. Bertwistle D, Fernandez M, Hawe E, Lorenzo M, Vickers A, Wolowacz S. Comparative efficacy and safety of interventions in the treatment of advanced soft tissue sarcoma: a systematic review and network meta-analysis. Poster PCN24, 19th Annual European Congress of the International Society for Pharmacoeconomics and Outcomes Research; Vienna, Austria. October 29-November 2, 2016.

23. Centers for Disease Control and Prevention. National Vital Statistics Report. Deaths: final data for 2013. Available at: http://www.cdc.gov/nchs/data_access/Vitalstatsonline.htm. Accessed May 7, 2016.

24. Reichardt P, Leahy M, García del Muro X, Ferrari S, Martín J, Gelderblom H, et al. Quality of life and utility in patients with metastatic soft tissue and bone sarcoma: the Sarcoma Treatment and Burden of Illness in North America and Europe (SABINE) study. Sarcoma. 2012;2012:740279.

25. Delea TE, Amdahl J, Nakhaipour HR, Manson SC, Wang A, Fedor N, et al. Cost-effectiveness of pazopanib in advanced soft-tissue sarcoma in Canada. Curr Oncol. 2014 Dec;21(6):e748-59.

26. Food and Drug Administration (FDA). Zinecard (dexrazoxane for injection) product label information. 2012. Available at: http://www.accessdata.fda.gov/drugsatfda_docs/label/2012/020212s013lbl.pdf. Accessed September 5, 2016.

27. Bui-Nguyen B, Ray-Coquard I, Chevreau C, Penel N, Bay JO, Coindre JM, et al.; GSF-GETO French Sarcoma Group. High-dose chemotherapy consolidation for chemosensitive advanced soft tissue sarcoma patients: an open-label, randomized controlled trial. Ann Oncol. 2012 Mar;23(3):777-84. doi: 10.1093/annonc/mdr282. Epub 2011 Jun 7. PubMed PMID: 21652583.

28. Judson I, Verweij J, Gelderblom H, Hartmann JT, Schöffski P, Blay J-Y, et al.; European Organisation and Treatment of Cancer–Soft Tissue and Bone Sarcoma Group. Results of a randomised phase III trial (EORTC 62012) of single-agent doxorubicin versus doxorubicin plus ifosfamide as first-line chemotherapy for patients with advanced, soft tissue sarcoma: a survival study by the EORTC Soft Tissue and Bone Sarcoma Group. Presented at the 2012 Connective Tissue Oncology Society Annual Meeting; Prague, Czech Republic. November 14-17, 2012.

29. Essential RBRVS. A comprehensive listing of RBRVS values for CPT and HCPCS codes. Utah: OptumInsight, Inc; 2016.

30. Eli Lilly and Company. Data on file. European medical record abstraction study. Project 2014-729 (RTI-HS Project 0303897). 2016.
